# Loss of the putative Rab GTPase, Ypt7, impairs the virulence of *Cryptococcus neoformans*

**DOI:** 10.3389/fmicb.2024.1437579

**Published:** 2024-07-25

**Authors:** Guanggan Hu, Xianya Qu, Kabir Bhalla, Peng Xue, Erik Bakkeren, Christopher W. J. Lee, James W. Kronstad

**Affiliations:** ^1^The Michael Smith Laboratories, University of British Columbia, Vancouver, BC, Canada; ^2^Department of Microbiology and Immunology, University of British Columbia, Vancouver, BC, Canada

**Keywords:** endomembrane trafficking, fungal pathogenesis, iron, heme, vacuole, cryptococcosis

## Abstract

Small GTPases of the Rab family coordinate multiple membrane fusion and trafficking events in eukaryotes. In fungi, the Rab GTPase, Ypt7, plays a critical role in late endosomal trafficking, and is required for homotypic fusion events in vacuole biogenesis and inheritance. In this study, we identified a putative *YPT7* homologue in *Cryptococcus neoformans*, a fungal pathogen causing life threatening meningoencephalitis in immunocompromised individuals. As part of an ongoing effort to understand mechanisms of iron acquisition in *C. neoformans*, we established a role for Ypt7 in growth on heme as the sole iron source. Deletion of *YPT7* also caused abnormal vacuolar morphology, defective endocytic trafficking and autophagy, and mislocalization of Aph1, a secreted vacuolar acid phosphatase. Ypt7 localized to the vacuolar membrane and membrane contact sites between the vacuole and mitochondria (vCLAMPs), and loss of the protein impaired growth on inhibitors of the electron transport chain. Additionally, Ypt7 was required for robust growth at 39°C, a phenotype likely involving the calcineurin signaling pathway because *ypt7* mutants displayed increased susceptibility to the calcineurin-specific inhibitors, FK506 and cyclosporin A; the mutants also had impaired growth in either limiting or high levels of calcium. Finally, Ypt7 was required for survival during interactions with macrophages, and *ypt7* mutants were attenuated for virulence in a mouse inhalation model thus demonstrating the importance of membrane trafficking functions in cryptococcosis.

## 1 Introduction

Iron is an essential nutrient involved in numerous biochemical processes including respiration, electron transport and the biosynthesis of amino acids, lipids and nucleotides. In host-microbe interactions, pathogens must sense and acquire iron, along with the other nutrients, to survive and proliferate in the hostile environment of the host. Free iron is scarce in mammalian hosts because it is sequestered by binding to transferrin, lactoferrin and other proteins (Kronstad et al., 2013; Murdoch and Skaar, 2022). Consequently, pathogens have evolved competitive systems to compete with the hosts for acquisition of iron and overcome nutritional immunity (Schaible and Kaufmann, 2004; Murdoch and Skaar, 2022). In *Cryptococcus neoformans*, a basidiomycetous fungal pathogen causing lethal meningoencephalitis in immunocompromised individuals (Casadevall, 2022), iron availability influences virulence factor elaboration including regulation of the polysaccharide capsule and melanization (Vartivarian et al., 1993; Jung et al., 2006; Xue et al., 2023a,b). Like other pathogenic fungi, *C. neoformans* acquires iron via cell surface reductases, exported reductants, cell wall melanin, and low and high affinity iron uptake systems (Jung et al., 2008, 2009; Cadieux et al., 2013; Kronstad et al., 2013; Horianopoulos and Kronstad, 2019; Xue et al., 2023a). We recently discovered that endomembrane trafficking plays an important role in iron use and, in particular, in the acquisition of iron from heme (Hu et al., 2013, 2015; Caza et al., 2018; Bairwa et al., 2019, 2020; Hu et al., 2021a; Xue et al., 2023a,b). Key components for heme use include the ESCRT complexes, clathrin-mediated endocytosis, and a cell-surface mannoprotein Cig1 (Cadieux et al., 2013; Hu et al., 2013, 2015; Bairwa et al., 2019). Similarly, the important pathogenic fungus *Candida albicans*, also acquires iron from hemoglobin via the endocytic pathway (Weissman et al., 2008).

Our understanding of the mechanisms of heme uptake and trafficking is incomplete for *C. neoformans*. We recently characterized the homotypic fusion and vacuole protein sorting (HOPS) tethering complex that is a key component of the endosomal pathway, mediating the fusion events between the late endosome and the vacuole (Bröcker et al., 2012). This complex consists of six subunits: Vam6 (Vps39), Vps11, Vps18, Vps16, Vps33 and Vps41 (Bröcker et al., 2012). In *C. neoformans*, mutants lacking *VPS41* or *VAM6* display defects in vacuolar morphology and endomembrane trafficking (Hu et al., 2021a,b). *VPS41* and *VAM6* are also required for robust growth on heme and inorganic iron sources, and a *vam6* mutant displays perturbed trafficking of iron acquisition functions (e.g., the high affinity iron permease Cft1) and impaired processing of the transcription factor Rim101, a regulator of heme acquisition. Vam6 and Vps41 are located at opposite ends of the HOPS complex and interact with Ypt7, a Rab7 GTPase present on late endosomes (Bröcker et al., 2012). However, it is unknown if Ypt7 is involved in iron use and other functions in *C. neoformans*.

Ypt7 is a member of the Rab family (Ras-associated binding protein, Rab GTPases) of the small GTPases. Rab GTPases coordinate intracellular membrane trafficking functions and act as regulatable molecular switches that recruit effector molecules when in their active GTP-bound form (Hutagalung and Novick, 2011). The conversion between GTP- and GDP-bound states is critical for regulating membrane fusion and trafficking events. During the conversion, Rab GTPases are involved in a cycle of attachment to and detachment from membranes (Hutagalung and Novick, 2011; Goody et al., 2017). Approximately 8 to 12 Rab GTPases have been identified so far in various fungal species, in comparison to at least 60 different Rab proteins in plants and mammals (Rutherford and Moore, 2002; Pereira-Leal, 2007; Liu et al., 2015). Connections between Rab GTPases and virulence in pathogenic fungi are emerging. For example, homologues of the *S. cerevisiae* Rab GTPase, Sec4/Sav1 are associated with protein secretion, vesicular trafficking, secondary metabolism and pathogenicity in a variety of pathogenic fungi. In *Fusarium verticillioides*, a plant pathogenic fungus causing ear rot and stalk rot on corn, Sec4 is critical in hyphal development, virulence, mycotoxin production and stress responses (Yan et al., 2020). In *Magnaporthe oryzae*, the rice blast fungus, disruption of MoSec4 resulted in defective secretion of extracellular proteins, abnormal hyphal development and reduced pathogenicity (Zheng et al., 2016).

The functions of Rab GTPases are starting to be characterized in *C. neoformans*. For example, one of the Rab GTPases, Sec4/Sav1, plays a role in exocytosis and trafficking of glucuronoxylomannan (GXM), the major cryptococcal capsule polysaccharide, within post-Golgi secretory vesicles (Yoneda and Doering, 2006). Conditional reduction of Sec4 levels results in temperature-sensitive growth and defective protein secretion, leading to accumulation of vesicles containing GXM at the septum and the bud. A recent investigation revealed that benzothioureas (BTUs) inhibit the late secretory pathway (post-Golgi), possibly through a direct interaction with Sav1/Sec4, and display highly selective fungicidal activity against *C. neoformans* (Beattie et al., 2020). These results suggest the potential for Rab GTPases to serve as drug targets for *C. neoformans*, and possibly also for other fungal pathogens. In the present study, we identified and characterized a Ypt7 homologue in *C. neoformans*. Ypt7 has conserved functions in vacuole biogenesis, intracellular membrane trafficking, and autophagy. The protein also plays a critical role in the elaboration and trafficking of virulence factors, the acquisition of iron from heme and is required for survival in macrophages and virulence in a mouse inhalation model.

## 2 Materials and methods

### 2.1 Fungal strains and culture conditions

Serotype A strain H99 (*C. neoformans* var. *grubii*) was employed as the wild-type (WT) strain. The WT strain and *ypt7* mutant derivatives were maintained on YPD medium (1% yeast extract, 2% peptone, 2% dextrose, and 2% agar). The nourseothricin, neomycin, and hygromycin resistance cassettes were from plasmids pCH233, pJAF1, and pJAF15, respectively (obtained from Dr. J. Heitman). YPD plates containing hygromycin (200 μg/mL) were used to select *ypt7* deletion transformants. Defined LIM and YNB (YNB with amino acids; adjusted to pH 7.0 with 1M 3-(N-Morpholino) propanesulfonic acid, MOPS plus 150 μM bathophenanthroline disulfonate (BPS) (YNB-LIM) were used as iron-limiting media. Iron-chelated dH_2_O was prepared by passage of dH_2_O through a column of Chelex-100 resin (BIORAD Chelex-100) and used to prepare YNB-BPS (Jung et al., 2009). Defined low-iron media (LIM) (0.5% glucose, 38 mM L-asparagine, 2.3 mM K_2_HPO_4_, 1.7 mM CaCl_2_·2H_2_O, 0.3 mM MgSO_4_·7H_2_O, 20 mM HEPES, 22 mM NaHCO_3_, 1 mL of 1,000X salt solution, 0.005 g/L CuSO_4_·5H_2_O, 2 g/L ZnSO_4_·7H_2_O, 0.01 g/L MnCl_3_·4H_2_O, 0.46 g/L sodium molybdate, 0.057 g/L boric acid) in iron-chelated dH_2_O adjusted to pH 7.4 with 0.4 mg/L sterile thiamine added post filtering) was prepared as described previously (Lian et al., 2005; Griffiths et al., 2012). YPD and/or YNB plates (YNB with amino acids) supplemented with different inhibitors or chemicals were used for phenotypic characterization. The minimal medium with low glucose for Aph1 induction was as follows: 0.1% glucose, 10 mM MgSO_4_, 0.5% KCl, 13 mM glycine, 3 μM thiamine, and 10 μM CuSO_4_. All chemicals were obtained from Sigma-Aldrich (St. Louis, MO) unless indicated otherwise. The strains employed in this study are listed in Supplementary Table S1.

### 2.2 Preparation of deletion constructs and deletion mutants

The *YPT7* gene was replaced with a hygromycin resistant cassette to obtain *ypt7* mutants. A *ypt7*::*hyg* deletion allele was constructed using a modified overlap PCR procedure (Davidson et al., 2002; Yu et al., 2004; Hu and Kronstad, 2010) and the primers listed in Supplementary Table S2. Briefly, the primers ypt7-1/ypt7-3 and ypt7-4/ypt7-6 were used with genomic DNA to obtain the left and right arms for the deletion construct. The selectable Hyg^r^ marker was amplified from the plasmid pJAF15 using the primers ypt7-2/ypt7-5. Overlap PCR was performed using the primers ypt7-1/ypt7-6 to yield a *ypt7::Hyg* allele that lacks the entire open reading frame of *YPT7* (1,129 bp). The resulting PCR product (3,280 kb) was used to transform strain H99 by biolistic transformation (Davidson et al., 2000). Transformants were screened by colony PCR with Extaq polymerase using the primers ypt7-7/ypt7-8 (negative screen) and ypt7-9/Hyg-po-F (positive screen). Primer ypt7-9 was designed from the region upstream of *YPT7*, and Hyg-po-F was designed for the HYG gene. Transformants in which the WT allele was replaced were confirmed by colony PCR analysis using ypt7-10/Hyg-Po-R (second positive screen). Two independent deletion mutants in the H99 background were designated the *ypt7-a4*, and *ypt7-b1* strains and were studied further. In addition, one *YPT7* mutant from the whole-genome deletion collection in the KN99 WT background, *ypt7-hm*, was included in some phenotypic assays for comparison. The *ypt7* deletion construct was also used to transform the Aph1-DsRed strain by biolistic transformation, and the positive transformants were employed for localization analysis of Aph1 (Lev et al., 2014).

### 2.3 Construction of a *GFP::YPT7* fusion allele

The N-terminus of the Ypt7 protein was tagged with green fluorescent protein (GFP) to examine the subcellular localization of the protein. Briefly, the upstream sequence (1,140 bp) of *YPT7* and the sequence of the *YPT7* coding region plus the terminator (2,283 bp) for the fusion construct were amplified from WT gDNA using the primer set Ypt7-pro-L1 and Ypt7-pro-R and the primer set GFP-Ypt7-L and ypt7-10-PO, respectively. The neomycin resistant cassette was amplified from the plasmid pJAF1 using primers P-Neo-marker-L and P-Neo-marker-R (1932 bp). The histone 3 promoter plus GFP gene were amplified from the plasmid pPZP-GFP-NATcc using primers H3GFP-L and H3GFP-R (1,316 bp). Overlap PCR was performed with the four PCR products as templates, using primers Ypt7-pro-L1 and ypt7-10-PO to yield the 6,671-bp construct. The construct was then used to transform the *ypt7* mutant strains (both *ypt7-a4* and *ypt7-b1*) by biolistic transformation. Following transformation, mutants were screened for resistance to G418, and the proper location and orientation of GFP were determined by PCR using primer pairs Ypt7GFP-screen-5R/Ypt7GFP-screen-3L, and Ypt7-Pro-L2/Neo-3L-long, respectively. One of positive transformants that complemented the phenotypes of *ypt7* deletion mutants, was used for further analysis, and also used as a complemented strain in phenotypic experiments. Primer sequences for all constructs are listed in Supplementary Table S2.

### 2.4 Construction of a *LYS4::mCherry* fusion allele

A modified overlapping PCR strategy was used to generate the constructs for tagging the C-terminal region of the Lys4 protein with mCherry. Briefly, the left and right arms for the Lys4-mCherry fusion construct were amplified from WT genomic DNA using the primer set Lys4-mCh-P1F and Lys4-mCh-P1R and the primer set Lys4-mCh-P3F and Lys4-mCh-P3R, respectively. The mCherry gene and the nourseothricin (NAT) resistance gene were amplified from the plasmid pGH024 using primers Lys4-mCh-P2F and Lys4-mCh-P2F. pGH024 was initially generated by cloning a BamHI and SpeI digested PCR fragment of mCherry gene from pLKB25 into BamHI and SpeI digested pCH233. Overlap PCR was performed using primers Lys4-mCh-P1F and Lys4-mCh-P3R to yield a 5-kb construct. The construct was then used to transform the GFP-Ypt7 strain by biolistic transformation. Transformants were screened for resistance to both NAT and G418, and the proper location and orientation of the gene fusions at the *LYS4* locus was determined by PCR.

### 2.5 Construction of a *GFP::Atg8* fusion allele

The N-terminus of Atg8 was tagged with GFP to examine the subcellular localization and stability of the protein. Briefly, the upstream sequence (1,006 bp) of *ATG8* and the sequence of *ATG8* coding region plus terminator (823 bp) for the fusion construct were amplified from WT gDNA using the primer set GFP-Atg8-P1L and GFP-Atg8-P1R and the primer set GFP-Atg8-P4L and GFP-Atg8-P4R, respectively. The nourseothricin (NAT) resistant cassette was amplified from the plasmid pCH233 using primers GFP-Atg8-P2L and GFP-Atg8-P2L (1,728 bp). The histone 3 promoter plus GFP gene were amplified from the plasmid pPZP-GFP-NATcc using primers GFP-Atg8-P3L and GFP-Atg8-P3R (1,325 bp). Overlap PCR was performed with the four PCR products as templates, using primers GFP-Atg8-P1L and GFP-Atg8-P4R to yield the 4,882-bp construct. The construct was then used to transform the WT and *ypt7* mutant strains (both *ypt7-a4* and *ypt7-b1*) by biolistic transformation. Following transformation, mutants were screened for resistance to NAT, and the proper location and orientation of GFP were determined by colony PCR using primer pairs Nat-3L-long/GFP-Atg8-P4Ra, and Nat-5R-long/GFP-Atg8-P1La, respectively.

### 2.6 Phenotypic assays

For serial dilution spot assays, overnight fungal cultures were washed twice in phosphate-buffered saline (PBS), and cell numbers were adjusted to 2 × 10^7^ cells/mL. Next, 10-fold serial dilutions were prepared, and 5 μL (covering a range of 10^5^ to 10^1^ cells) was spotted onto agar medium. Plates were then incubated at 30°C or 37°C, or 39°C for 2 days before being photographed. Capsule formation was examined by differential interference contrast (DIC) microscopy after incubation for 24–48 h at 30°C in defined LIM and staining with India ink. Melanin production was examined on L-3,4-dihydroxyphenylalanine plates containing 0.1% glucose.

To assess the response of *C. neoformans* WT, *ypt7*, and *YPT7* complemented strains to various stress conditions, exponentially growing cultures were washed, resuspended in H_2_O, and adjusted to a concentration of 2 × 10^7^ cells/mL. The cell suspensions were diluted 10-fold serially, and 5 μL of each dilution was spotted onto YPD and/or YNB plates supplemented with different compounds. Plates were incubated for 2–10 days at 30°C or 37°C and photographed. The responses of strains to osmotic stress, and agents that challenge cell wall integrity were examined. Sensitivity to trafficking inhibitors brefeldin A (BFA) and monensin were examined by spotting the cell dilutions on YPD containing 30 μg/mL of BFA and 625 μg/mL of monensin. The antifungal drugs fluconazole (5 μg/mL) and miconazole (0.2 μg/mL) were also tested.

### 2.7 Measurement of extracellular acid phosphatase activity

The WT strain, *ypt7* mutants, and the complemented strain were grown overnight in YPD medium. The cells were harvested, washed with distilled water twice, and resuspended in either MM-250 Pi (the MM medium supplemented with 250 mM KH_2_PO_4_), or MM-0 Pi (MM without addition of the phosphate salt) at a concentration of OD_600_ = 2. The cells were further incubated at 30°C for 3 h, before harvesting by centrifugation. Fifty microliters of supernatant of each sample was collected and measured for acid phosphatase activity using Acid Phosphatase Assay Kit from Sigma Aldrich.^1^ Reactionmixtures were incubated at 37°C and 200 μL of saturated 0.5 M NaOH was added to the mixtures at different time points (10 min, 30 min and 60 min) to stop the reaction. The extent of 4-nitrophenyl phosphate hydrolysis was measured spectrophotometrically at 405 nm. The assay was performed in triplicate and repeated three times.

### 2.8 Protein extraction and immunoblot analysis

The cells were grown overnight at 30°C to late logarithmic phase in 50 mL of YPD medium, diluted 1 in 10 in fresh YPD, and grown in a final volume of 50 mL for 4 h with shaking. For nitrogen starvation treatment, the cells were harvested, washed and resuspended in MM-N, and further incubated at 30°C for the indicated time intervals. Protein extracts were obtained with a modified lysis buffer 50 mM Tris-HCl pH7.5, 5 mM EDTA, 100 mM NaCl, 1% Triton X-100, and 1X EDTA-free protease inhibitor cocktail (Roche, Basel, Switzerland). Protein concentration was determined using the PierceTM BCA Protein Assay kit following the manufacturer’s instructions (Thermo Fisher, Waltham, MA, United States). For all immunoblot analyses, proteins were transferred onto PVDF (GE Healthcare, Boston, MA, United States) using a wet transfer at 80 V for 1 h. Membranes were blocked in Tris-buffered saline with Tween 20 (TBST) with 5% skim milk and incubated with the following antibodies at the indicated concentrations: monoclonal anti-GFP (Santa Cruz Biotechnology, SCBT) at 1:1,000, and anti-histone H3 (Millipore Sigma, Oakville, ON, CA), and anti-mouse HRP (Bio-Rad, Hercules, CA, United States) at 1:5,000. Immunoblots were visualized using chemiluminescence (GE Healthcare, Boston, MA, United States).

### 2.9 Macrophage survival assays

The effect of *YPT7* deletion on fungal survival during incubation with macrophages was assessed as previously described (Hu and Kronstad, 2010; Caza et al., 2016; Hu et al., 2021a). Briefly, the murine macrophage-like cell line J774A.1 was maintained at 37°C in 5% CO_2_ in Dulbecco’s modified.

Eagle’s medium supplemented with 10% heat-inactivated fetal bovine serum, 1% nonessential amino acids, 100 μg/mL penicillin-streptomycin, and 4 mM L-glutamine (Invitrogen). The cell line was used between passages 5 and 10. Cells of the WT, two *ypt7*, mutants, and the *YPT7* complemented mutant were opsonized with monoclonal antibody 18B7 against capsule (10 μg/mL; a generous gift from Dr. Arturo Casadevall), and macrophages were stimulated with 150 ng/mL phorbol myristate acetate (PMA) for 2 h prior to coincubation at a multiplicity of infection of 1:1. Macrophages were inoculated at 1 × 10^5^ cells and washed after 2 h of inoculation to remove unattached, extracellular fungal cells. After 24 h of incubation, sterile, ice-cold distilled H_2_O was applied to each well to lyse the macrophages (confirmed microscopically). Fungal growth was measured by plating cells on YPD and determining CFUs. The assay was performed in triplicate for each strain, and the experiment was repeated three times with consistent results. Student’s *t*-test was used to determine the statistical significance of the differences in fungal survival.

### 2.10 Assessment of virulence in a murine model

Female BALB/c mice, 4–6 weeks old, were obtained from Charles River Laboratories (Pointe-Claire, Quebec, Canada) and used in an inhalation model of cryptococcosis. A cell suspension of 2 × 10^5^ cells in a 50 μL volume was used for intranasal instillation, and 10 mice were inoculated per strain. The status of the mice was monitored once per day post-inoculation. Mice reaching the humane end point were euthanized by isoflurane overdose followed by CO_2_ asphyxiation. The protocol for the virulence assay (protocol A21-0105) was approved by the University of British Columbia Committee on Animal Care. For determination of the fungal load in organs, infected mice were euthanized by CO_2_ inhalation and organs were excised, weighed, and homogenized in 2 volumes of phosphate-buffered saline using a MixerMill (Retsch, Cole-Parmer, Montreal, Canada). Serial dilutions of the homogenates were plated on YPD plates containing 50 μg/mL chloramphenicol, and colony-forming units were counted after an incubation for 48 h at 30°C. Differences in virulence were statistically assessed by log-rank tests for survival and by using the two-tailed nonparametric Mann Whitney U test from the GraphPad Prism 7 program (GraphPad Software, San Diego, CA). For histological analyses the lung tissues were stained with hematoxylin and eosin (H&E) and mucicarmine.

## 3 Results

### 3.1 Identification of Ypt7 in *Cryptococcus neoformans*

The HOPS complex is important for heme use and virulence in *C. neoformans*, and Ypt7 is the Rab GTPase required for HOPS functions and vacuole biogenesis (Haas et al., 1995; Bröcker et al., 2012; Auffarth et al., 2014; Karim et al., 2018; Hu et al., 2021a). We initially used the *S. cerevisiae* Ypt7 amino acid sequence and BLASTp to identify candidate Ypt7 orthologs as a prelude to functional characterization. A gene (CNAG_02575) was identified that encoded a candidate Ypt7 protein of 206 amino acids with a CXC (C = cysteine, X = any amino acid-farnesylated cysteine) motif at the carboxyl terminus. The post-translational prenylation of the CXC motif mediates the membrane localization of Ypt7 proteins. The candidate Ypt7 amino acid sequence displayed conserved sequence motifs of the Ras superfamily, including Rab family motifs (RabF), Rab subfamily motifs (RabSF) and 5 G-Boxes (G1-G5). Multiple sequence alignments revealed that Ypt7 from *C. neoformans* shares high amino acid sequence identity with Ypt7 from other organisms including *Ustilago maydis* (87%), *S. pombe* (71%), *S. cerevisiae* (65%), *Mus musculus* (78%), and *Homo sapiens* (78%) (Supplementary Figure S1). In a larger survey, we identified a total of 10 putative Rab GTPases in *C. neoformans*, including the Ypt7 ortholog and the Rab GTPase, Sec4/Sav1, that functions in the secretion of capsule polysaccharide via exocytosis (Yoneda and Doering, 2006) (Supplementary Table S3). The other candidate Rab GTPases have not been characterized in *C. neoformans*. Overall, the sequence analysis supports the conclusion that CNAG_02575 encodes a Ypt7 ortholog in *C. neoformans*.

### 3.2 Ypt7 has a conserved role in vacuole fusion in *Cryptococcus neoformans*

**Figure 1:**
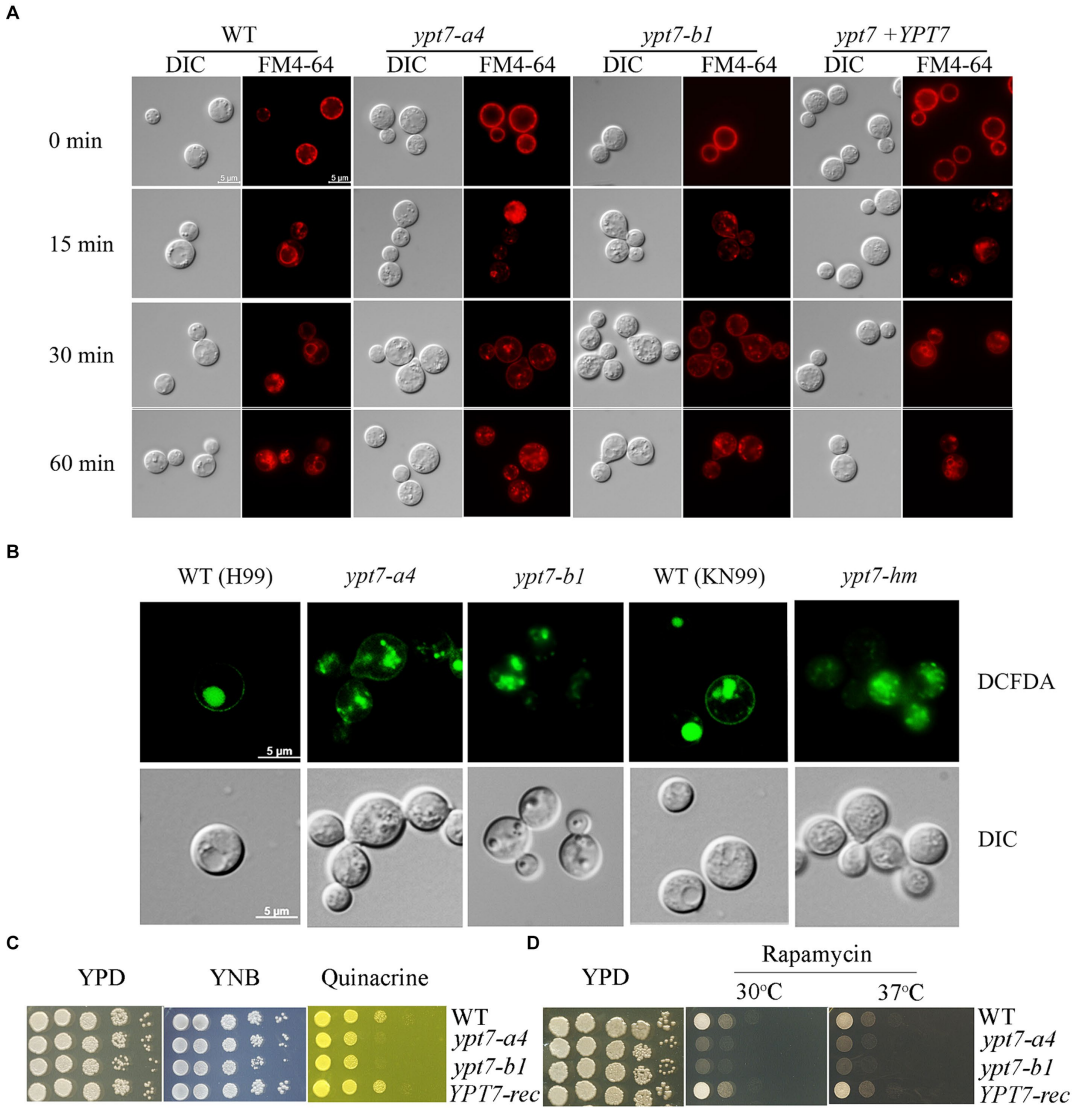
Vacuolar morphology and function in *ypt7* mutants. **(A)** Analysis of endocytosis by FM4-64 internalization. The cells of WT, two independent *ypt7* mutants (*ypt7-a4* and *ypt7-b1*), and the complemented strain grown in YPD were stained with 5 μM FM4-64 and observed by fluorescence microscopy at the indicated times. Bar = 5 μm. **(B)** Vacuoles of WT (H99 or KN99) or *ypt7* mutant cells were stained with 5-(and-6)-carboxy-20,7-dicholorofluorescein diacetate (carboxy-DCFDA). Bar = 5 μm. **(C,D)** Growth defects of *ypt7* deletion mutants on quinacrine **(C)**, or rapamycin **(D)**. Ten-fold serial dilutions of each strain were spotted on the indicated media, and the plates were incubated at indicated temperatures for 2 days before being photographed. The assays were repeated at least three times.

Ypt7 interacts with the HOPS complex to mediate endosome - vacuole fusion, and our previous study revealed that loss of the HOPS complex subunits, Vam6 and Vps41, led to fragmented vacuoles and defective endocytic trafficking (Hu et al., 2021a). We therefore tested whether loss of Ypt7 caused similar phenotypes with regard to vacuole morphology, consistent with roles in endocytosis and vacuolar function. We generated two independent *YPT7* deletion mutants, *ypt7-a4* and *ypt7-b1*, and a complemented mutant, and then used the endocytic dye FM4-64 to examine endocytic trafficking and vacuolar staining by fluorescence microscopy (Figure 1A). Deletion of *YPT7* did not influence cell morphology, and accumulation of FM4-64 on endosomes and vacuolar membranes was detected starting at 15 to 30 min after staining for all strains. However, loss of Ypt7 resulted in multiple, small fragmented vacuoles in the cytoplasm, similar to the B or C vacuolar phenotypes in *S. cerevisiae*, and as previously observed in the *vam6* or *vps41* mutants in *C. neoformans* (Hu et al., 2021a). Staining with the vacuole-sequestered dye c-DCFDA also revealed fragmented vacuoles in the mutants, consistent with the findings with FM-4-64 (Figure 1B). For confirmation, we analysed an independent deletion mutant, designated *ypt7-hm* in the background of the WT strain KN99 from the deletion collection (Liu et al., 2008) and observed multiple, small fragmented vacuoles, similar to those in *ypt7* mutants in H99 background (Figure 1B). Inspection of ~100 cells per strain revealed that >95% of the vacuoles had a distinct and normal morphology in the WT and reconstituted cells, but less than 5% were normal and instead were fragmented in the *ypt7-a4* or *ypt7-b1* mutant cells. The strains were also stained with the lipophilic vacuolar membrane dye MDY64 and disorganized vacuole structures were observed as with FM-4-64 (Supplementary Figure S2). Overall, these observations support the hypothesis that Ypt7 is required for the homotypic fusion of vacuoles.

We next examined vacuolar function by testing mutant sensitivity to quinacrine and rapamycin. Quinacrine is an antimalarial drug that accumulates in vacuoles, and we found that deletion of *YPT7* resulted in increased sensitivity to the drug, compared to the WT and complemented strains (Figure 1C). The antibiotic rapamycin targets the TOR pathway that regulates cell metabolism and proliferation (Heitman et al., 1991). Similar to the other class C *vps* mutants in *S. cerevisiae*, the HOPS complex mutant *vam6* is unable to recover from rapamycin-induced growth arrest (Zurita-Martinez et al., 2007; Hu et al., 2021a). Similarly, the *ypt7* mutants displayed defective growth in the presence of rapamycin at both 30°C and 37°C, and the *YPT7* complemented strain restored growth to the WT level (Figure 1D). Collectively, these results support the conclusion that the candidate Ypt7 protein in *C. neoformans* plays conserved roles in vacuole biogenesis and functions, a finding consistent with participation in endomembrane trafficking similar to the HOPS complex components, Vam6 and Vps41.

### 3.3 Ypt7 is localized on the vacuolar membrane and vCLAMPs, and influences mitochondria functions

**Figure 2:**
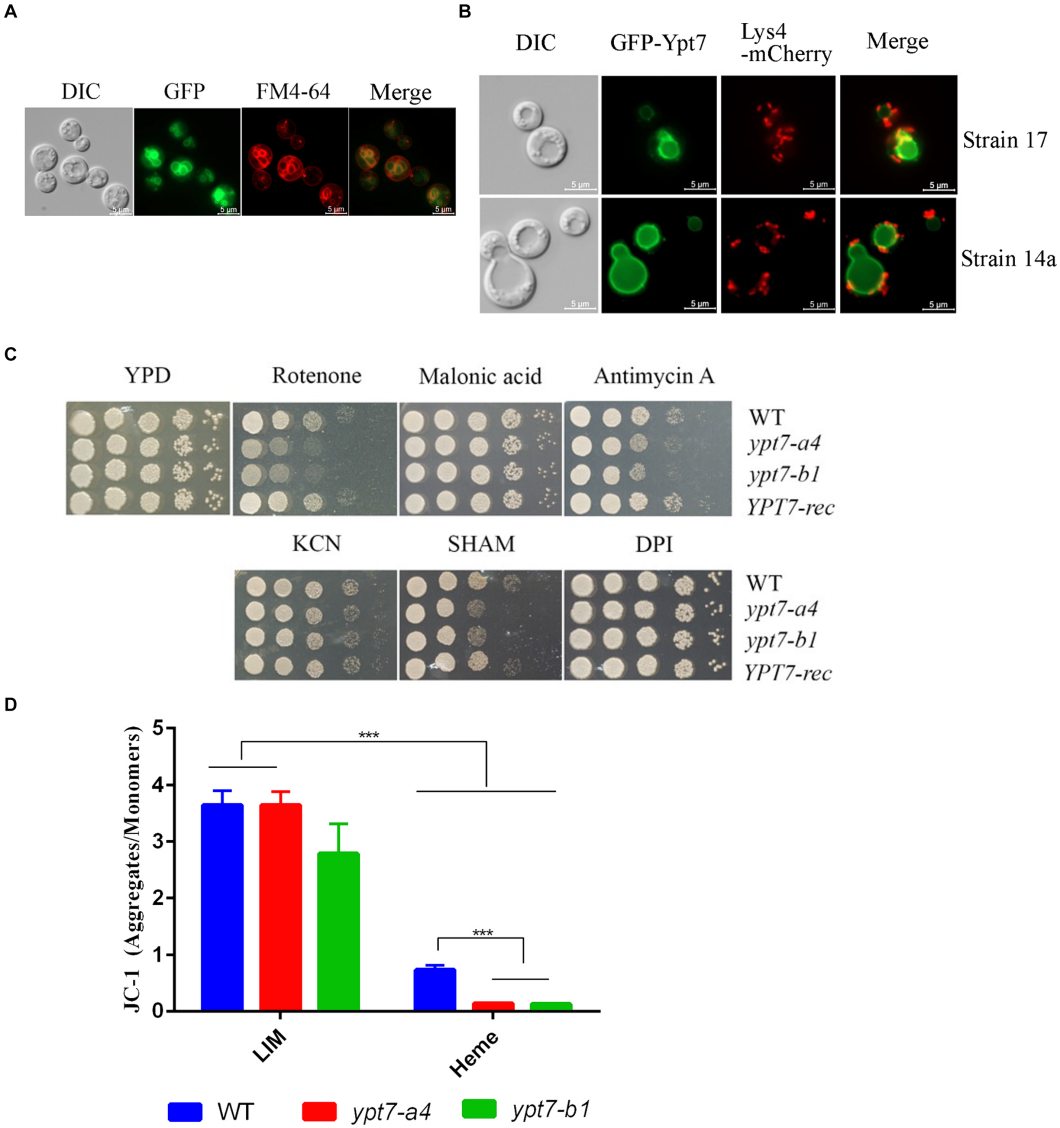
Ypt7 localizes to the vacuolar membrane and vacuolar-mitochondria contact sites, and contributes to mitochondrial function. **(A)** A strain in which Ypt7 was tagged with GFP at the N-terminus was grown in YPD medium for 24 h and stained with FM4-64 to reveal the vacuolar membrane. Bar = 5 μm. **(B)** Co-localization of GFP-Ypt7 with the mitochondrial protein, Lys4, tagged with mCherry at the C-terminus. Bar = 5 μm. **(C)** Spot assays on YPD medium containing inhibitors of electron transport chain complexes I to IV and the alternative oxidase. The following concentrations were used: 75 μg/mL rotenone, 2 mM malonic acid, 5 μg/mL antimycin A, 10 mM potassium cyanide (KCN), 10 mM salicylic hydroxamate (SHAM), and 50 μM diphenyleneiodonium (DPI). The plates were incubated at 30°C for 2 days before being photographed. **(D)** Flow cytometry analysis of mitochondria membrane potential in the indicated strains. Measurements were performed on cells grown on low iron YNB (YNB-LIM) with or without hemin (100 μM) at 30°C for 16 h with staining with the mitochondrial dye JC-1 (5,5′,6,6′-tetrachloro-1,1′,3,3′-tetraethylbenzimi-dazolylcarbocyanine iodide, 2.5 μM) for 30 min at 30°C. The data represent the averages from three independent experiments ± standard errors of the means (SEMs). Statistical significance was determined by ANOVA followed by uncorrected Fisher’s LSD test (^***^p < 0.001).

We next generated strains carrying a GFP-Ypt7 fusion in the independent *ypt7* mutants (*ypt7-a4* and *ypt7-b1*) to examine cellular localization. The Ypt7 promoter was originally used to transcribe the GFP-Ypt7 fusion protein, but the resulting transformants all exhibited weak GFP fluorescence. Therefore, a constitutive histone 3 promoter was employed to generate a stronger signal. Importantly, the *in vitro* phenotypes of the *ypt7* mutants were restored to WT in the resulting GFP-Ypt7 strains, indicating that the fusion protein was functional; the GFP-Ypt7 strain was included as a *YPT7*-reconstituted strain in some of the phenotypic analyses. The fluorescent GFP-Ypt7 signal overlapped with the ring-like structure of the vacuolar membrane, and with the internal membranes stained with FM-4-64 (Figure 2A). We also determined whether defects in genes encoding CORVET or HOPS complex proteins influenced the localization of Ypt7. Specifically, we deleted *VPS8* or *VPS3* (CORVET complex), or *VPS41* or *VAM6* (HOPS complex) in the GFP-Ypt7 strain. Loss of Vps3 or Vps8 did not influence the localization of GFP-Ypt7 with regard to vacuolar structure. However, the mutants with deletions in *VPS41* or *VPS39* displayed fragmented vacuoles and the fluorescent signal of GFP-Ypt7 was dispersed in the cytoplasm (Supplementary Figure S3), indicating a significant impact of HOPS complex on localization of Ypt7.

Ypt7 plays an essential role in the membrane contact sites between the vacuole and mitochondria (vCLAMP) in *S. cerevisiae* (Hönscher et al., 2014). We therefore examined the association between Ypt7 and mitochondria by comparing the GFP-Ypt7 signal with that of the Lys4 mitochondrial protein tagged with mCherry. We previously found that Lys4 is localized on the mitochondrial membrane in *C. neoformans* (Do et al., 2016). Here we found that Lys4-mCherry localized to mitochondria as expected, and we observed the co-localization of Lys4-mCherry and GFP-Ypt7 at putative vCLAMP regions (Figure 2B). We further verified the finding by staining the GFP-Ypt7 cells with mitotracker (Supplementary Figure S4). These results indicated that Ypt7 is associated in part with vCLAMP sites and may participate in mitochondria—vacuole interactions.

The localization of GFP-Ypt7 with vCLAMP sites prompted a further exploration of the role of Ypt7 in mitochondrial functions. We therefore analyzed the sensitivity of *ypt7* mutants to electron transport chain inhibitors (ETC) including rotenone, malonic acid, antimycin A, potassium cyanide [KCN], salicylic hydroxamate (SHAM), and diphenyleneiodonium (DPI) (Chaban et al., 2014; Genova and Lenaz, 2014; Caza et al., 2016) (Figure 2C). The assays revealed that loss of *YPT7* caused impaired growth on medium supplemented with rotenone, antimycin A, or SHAM, and the *YPT7* reconstituted strain restored the growth to the WT level (Figure 2C). Specifically, rotenone is an inhibitor of complex I, antimycin A of complex III, while SHAM targets to the alternative oxidase of ETC. Moreover, deletion of *YPT7* caused a significant reduction in mitochondrial membrane potential, in particular when heme was added as the sole iron source, as revealed by staining with JC-1 and flow cytometry (Figure 2D). Taken together, this evidence corroborates the hypothesis that Ypt7 influences mitochondrial-related functions.

### 3.4 Loss of Ypt7 confers sensitivity to ER stress and impairs membrane integrity

**Figure 3:**
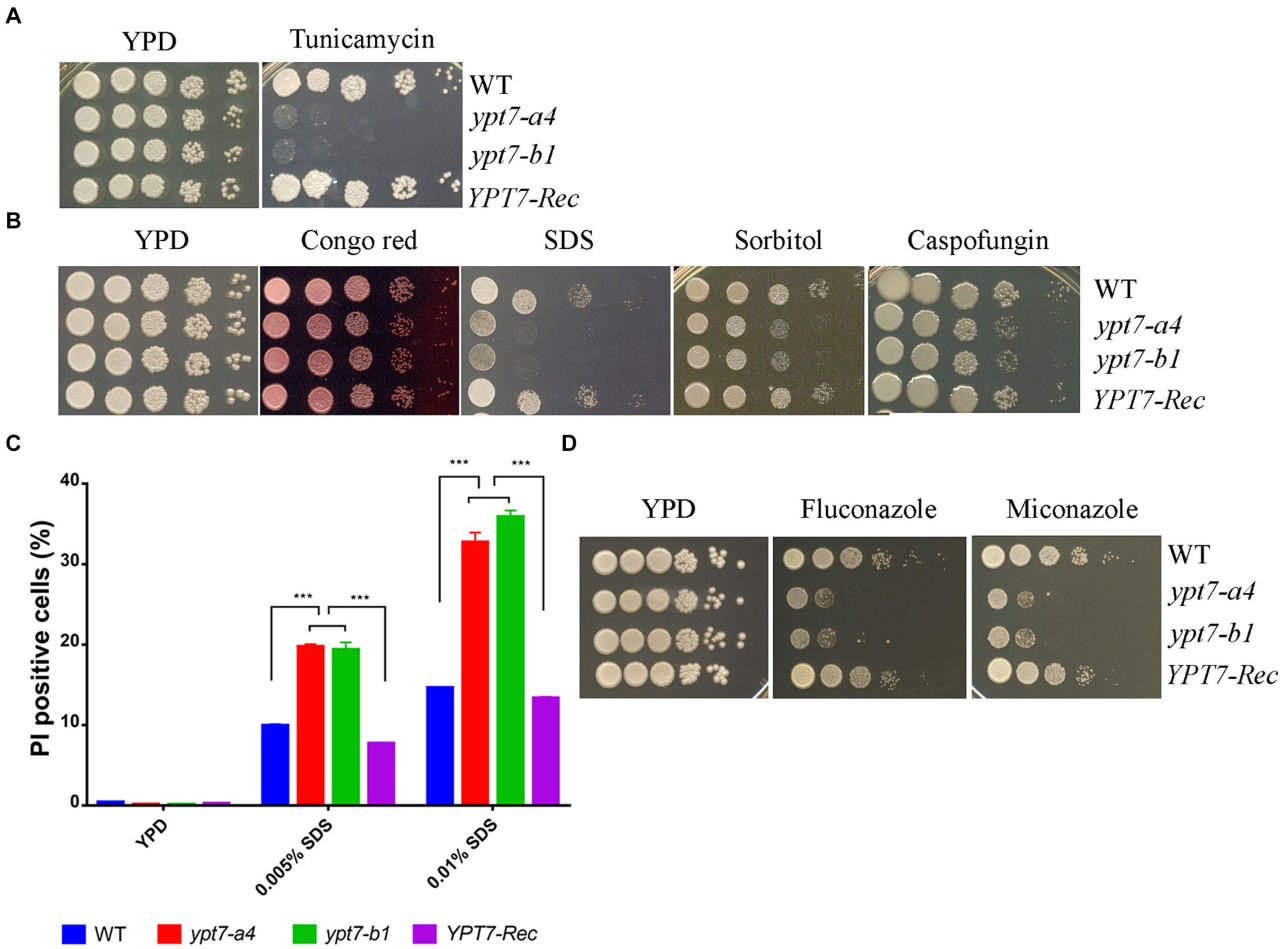
Ypt7 regulates the response to ER stress and cell membrane integrity. **(A)** Spot assays were performed with the WT strain, the *ypt7* deletion mutants, the *YPT7* complemented strain on YPD supplemented with 250 ng/mL tunicamycin. The plates were incubated at 30°C for 2 days before photographed. **(B)** Spot assays with the strains on YPD medium containing 0.5 mg/mL Congo Red, 0.01% SDS, 1.2 M sorbitol or 10 μg/mL caspofungin. The plates were incubated for 2 days before photographed. **(C)** Flow cytometry analysis of cell membrane permeability of the indicated strains upon treatment with either 0.005% or 0.01%. The measurements were obtained using staining for non-viable, permeable cells with the dye propidium iodide (PI, 2.5 μg/mL) for 30 min at 30°C. Data represent the averages from three independent experiments ± standard deviations (SD). Statistical significance was determined by ANOVA (^***^p < 0.001) in GraphPad V6. **(D)** Spot assays to test sensitivity to fluconazole (5 μg/mL) and miconazole (0.2 μg/mL). The plates were incubated at 30°C for 2 days before being photographed. The assays were repeated at least three times.

We next examined the sensitivity of the *ypt7* mutants to ER stress and cell-wall or membrane damaging agents. Notably, loss of Ypt7 impaired growth in the presence of tunicamycin, an inhibitor of glycoprotein synthesis that induces the unfolded protein response, thus indicating a role of Ypt7 in the response to ER stress (Figure 3A). The complemented strain grew as well as the WT strain on tunicamycin. Further assays revealed that the *ypt7* mutants were not impaired for growth on calcofluor white and caffeine (agents that challenge cell wall integrity), but displayed subtly impaired growth on Congo red, and markedly reduced growth on SDS (Figure 3B). These results suggested altered cell wall and membrane integrity in the *ypt7* mutants, and we therefore examined the sensitivity of the strains to caspofungin, a drug targeting the fungal cell wall glucan synthesis. *C. neoformans* is generally insensitive to this drug (Gerik et al., 2005) but loss of Ypt7 caused sensitivity (Figure 3B). In addition, we found that the mutants exhibited significant reduced growth on YPD medium with sorbitol, a reagent (at 1 M) that causes osmotic stress (Figure 3B).

The marked sensitivity to SDS prompted a more detailed examination of membrane integrity by flow cytometry analysis. In this case, staining with propidium iodide (PI) revealed that the cell membrane permeability was compromised in the *ypt7* mutants (Figure 3C). Specifically, the mutants exhibited higher percentages of PI staining after treated with both concentrations of SDS (either 0.005% or 0.01%) compared with the WT strain, indicating increased permeability (Figure 3C). The defect in membrane integrity in the mutants prompted an additional examination of sensitivity to azole drugs that disrupt the synthesis of ergosterol, the principal sterol in fungal cell membranes. As expected, the *ypt7* mutants displayed hypersensitivity to either fluconazole or miconazole (Figure 3D). Together, the data provide additional evidence that Ypt7 is involved in maintenance of membrane integrity.

### 3.5 Mutants lacking Ypt7 are sensitive to calcium and calcineurin inhibitors

**Figure 4:**
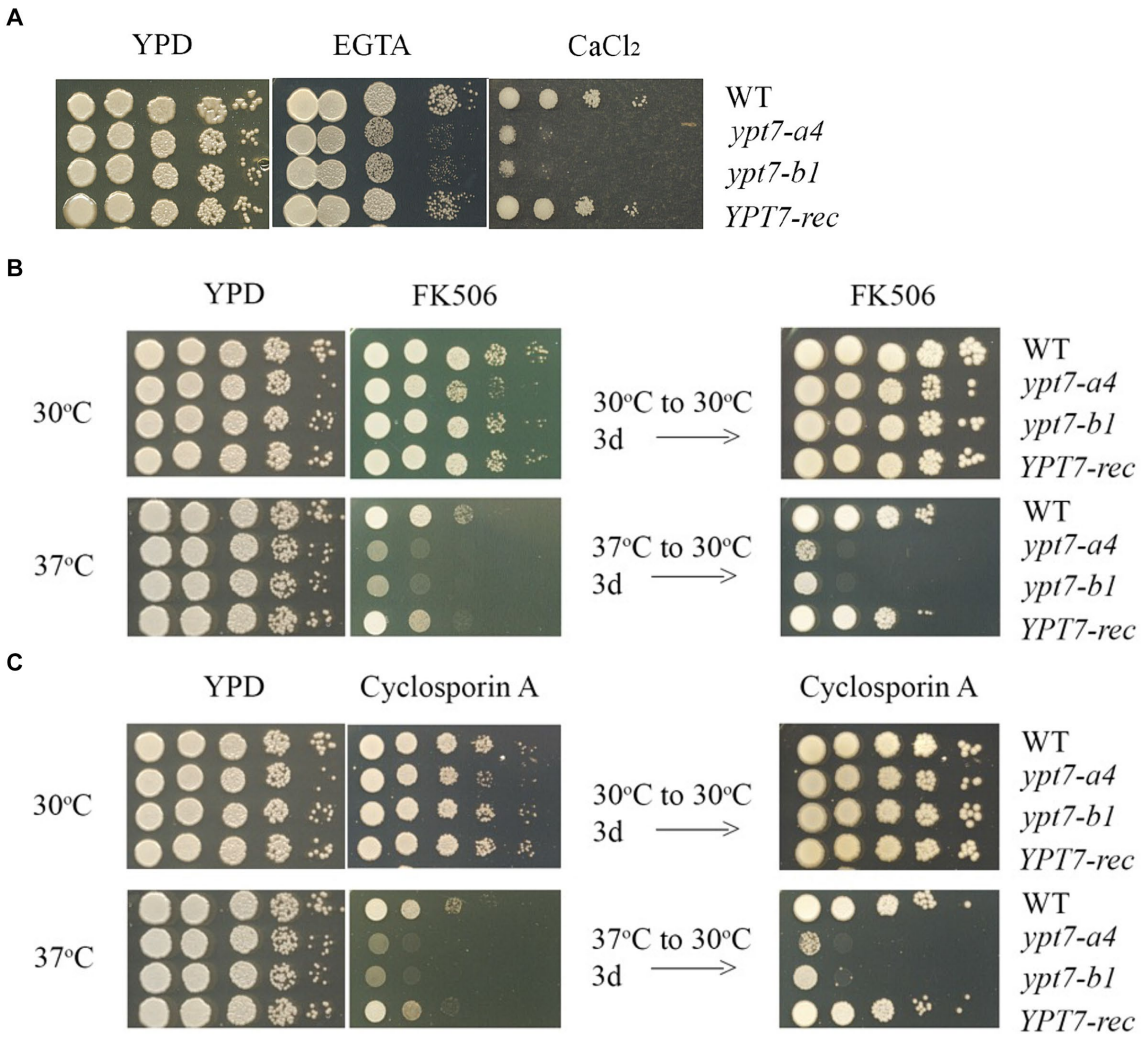
Ypt7 regulates thermotolerance and interacts with the calcineurin pathway. **(A)** The WT strain, two independent *ypt7* mutants (labeled *ypt7-a4* and *ypt7-b1*) and the *YPT7* complemented strain were grown overnight at 30°C. Ten-fold serial dilution of the cells were spotted on YPD with or without excess calcium chloride (400 mM) or the calcium chelator EGTA (5 mM, ethylene glycol-bis (b-aminoethyl ether)-N, N, N′, N′-tetraacetic acid). The plates were incubated at 30°C for 2 days before being photographed. **(B)** Ten-fold serial dilutions of the cells were spotted on YPD supplemented with 1 mg/mL of FK506 and incubated for 2 days at 30°C or 37°C. The plates were then transferred to 30°C for 3 days before being photographed. **(C)** The experiment in **(B)** was performed in parallel with YPD supplemented with 100 mg/mL of cyclosporin A. The assays were repeated at least three times.

In *C. neoformans* and the other pathogenic fungi, calcineurin influences hypersensitivity to a variety of stress conditions including agents that perturb cell wall and membrane integrity, elevated temperature, oxidative and ionic stress, and ER stress (Park et al., 2016, 2019; Chow et al., 2017; Fu et al., 2018; Stempinski et al., 2022). Given that the *ypt7* mutants shared these phenotypes, we tested the sensitivity of the strains to calcium and inhibitors of calcineurin signaling. The *ypt7* mutants exhibited hypersensitivity to either excess (200 or 400 mM CaCl_2_), or limiting calcium (5 or 10 mM EGTA) thus implicating Ypt7 in calcium homeostasis (Figure 4A). Given these results, we hypothesized that the *ypt7* mutants would be hypersensitive to inhibitors of calcineurin signaling. Tacrolimus (FK506) and cyclosporine A (CsA) are immunosuppressive, antifungal drugs, that bind to the immunophilins FKBP12 and cyclophilin, respectively, to inhibit calcineurin (Park et al., 2016; Chow et al., 2017; Fu et al., 2018). We tested the strains on either 1 μg/mL FK506 or 100 μg/mL CsA, with incubation at either 30°C or 37°C for 2 days before transfer to 30°C for additional 3 days (to test survival). As shown in Figures 4B,C, the *ypt7* mutants had subtly reduced growth after 2 days at 30°C, but grew as robustly as the WT and the complemented strains after an additional 3 days of incubation. The growth of all strains was inhibited with either 1 μg/mL FK506 or 100 μg/mL CsA after 2 days of incubation at 37°C. Although the *ypt7* mutants generally grew as well as the WT and the complemented strains on YPD at 37°C, the *ypt7* mutants displayed more markedly impaired growth of FK506 and CsA (Figures 4B,C). Transfer of the plates with either FK506 or CsA after 2 days at 37°C to 30°C for an additional 3 days demonstrated drastically reduced survival of the *ypt7* mutants. Overall, the analysis supports the hypothesis that Ypt7 influences thermotolerance, at least in part, via calcineurin signaling.

### 3.6 Ypt7 influences intracellular trafficking and secretion

**Figure 5:**
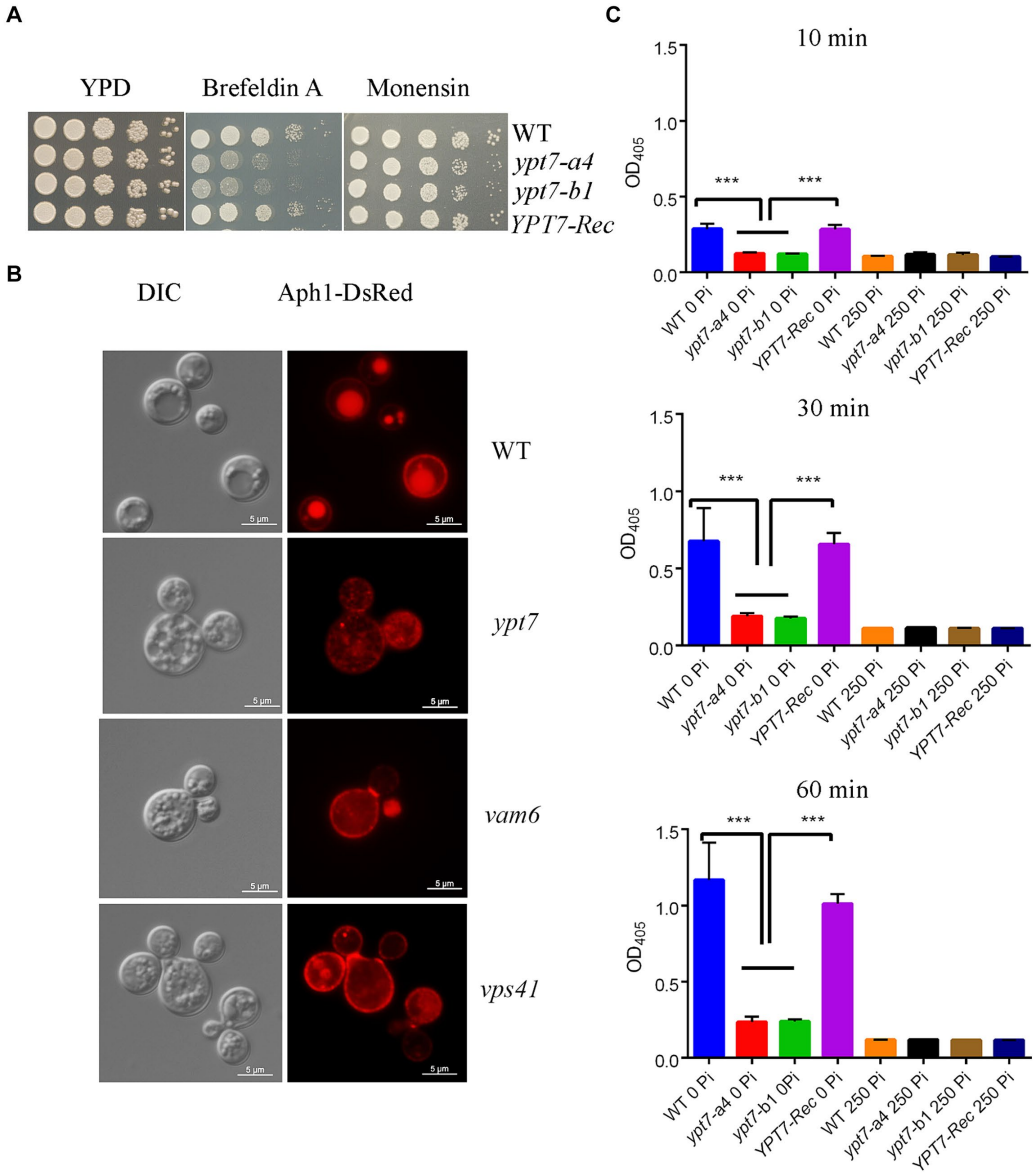
Loss of Ypt1 impairs intracellular trafficking, mislocalizes Aph1 and decreases acid phosphatase activity. **(A)** Spot assays were performed with the WT strain, the *ypt7* deletion mutants, the *YPT7* complemented strain on YPD supplemented with 20 μg/mL brefeldin A (BFA) or 625 ng/mL monensin. The plates were incubated at 30°C for 2 days before being photographed. **(B)** Analysis of the localization of DsRed-tagged Aph1 (acid phosphatase, Aph1-DsRed) at the cell periphery and vacuoles in the WT strain and at the cell periphery and cytoplasm in *ypt7*, *vam6* and *vps41* mutants. Significantly reduced fluorescence is present in fragmented vacuoles in mutants. Bar = 5 μm. DIC, differential interference contrast. **(C)** Measurement of extracellular acid phosphatase activity in WT, two *ypt7* mutants and the complemented strain. Cells were cultured in minimum medium with or without 250 mM KH_2_PO_4_ before activity measurements. The extent of 4-nitrophenyl phosphate hydrolysis was measured spectrophotometrically at 405 nm at three different time points (10 min, 30 min and 60 min). The assays were repeated for three times. Data represent the averages from three independent experiments ± standard deviations (SD). Statistical significance was determined by ANOVA (^***^p < 0.001) in GraphPad V6.

We next tested the growth of *ypt7* mutants on YPD medium supplemented with inhibitors that influence trafficking. Interestingly, the mutants exhibited markedly reduced growth on brefeldin A (BFA) but subtly reduced growth on monensin indicating a likely function of Ypt7 in ER-Golgi intracellular trafficking (Figure 5A). BFA inhibits the anterograde transport of proteins between the ER and the Golgi apparatus, and monensin is a Na/H ionophore that blocks intracellular transport in both the trans-Golgi and post-Golgi compartments (Hu et al., 2007). We further investigated the role of Ypt7 in intracellular trafficking and secretion by examining the localization of the extracellular acid phosphatase Aph1. Several pathogenic fungi including *C. albicans*, *Aspergillus fumigatus*, and *C. neoformans* secrete extracellular acid phosphatase (Bernard et al., 2002; Collopy-Junior et al., 2006; Vidotto et al., 2006; Portela et al., 2010; Lev et al., 2014). In *C. neoformans*, Aph1 contributes to virulence, and DsRed-tagged Aph1 is transported to the cell periphery and vacuoles via endosome-like structures and is enriched in bud necks in the low-phosphate condition (Lev et al., 2014). We found that Aph1-DsRed is visible in the cell periphery and in bud necks for WT and *ypt7* mutant cells grown in low-phosphate medium. In WT cells, Aph1-DsRed was largely enriched in vacuoles, while the protein was discernable in the multiple fragmented vacuoles in *ypt7* mutants (Figure 5B). We extended our analysis by examining Aph1-DsRed localization in the HOPS complex mutants (*vam6* and *vps41*), and found that Aph1-DsRed was mainly localized to the fragmented vacuoles, similar to the result for the *ypt7* mutants. These findings suggest deletion of *YPT7*, *VAM6*, or *VPS41* influences the localization of Aph1-DsRed, consistent with a role in protein trafficking.

We extended our analysis on acid phosphatase production and secretion by measuring the extracellular acid phosphatase activity in the WT, *ypt7* mutant and complemented strains. The cells were grown in medium supplemented with or without phosphate (250 mM KH_2_PO_4_) for 3 h and extracellular acid phosphatase activity was measured at 10, 30 and 60 min after incubation with the substrate (Figure 5C). The activity increased with time and addition of phosphate inhibited the activity in all strains. As expected, deletion of *YPT7* led to reduced extracellular acid phosphatase activity, compared with either WT or the complemented strains. We conclude that Ypt7 and the components of the HOPS complex function in intracellular trafficking, and the production and secretion of extracellular acid phosphatase.

### 3.7 Autophagy is impaired upon loss of Ypt7

**Figure 6:**
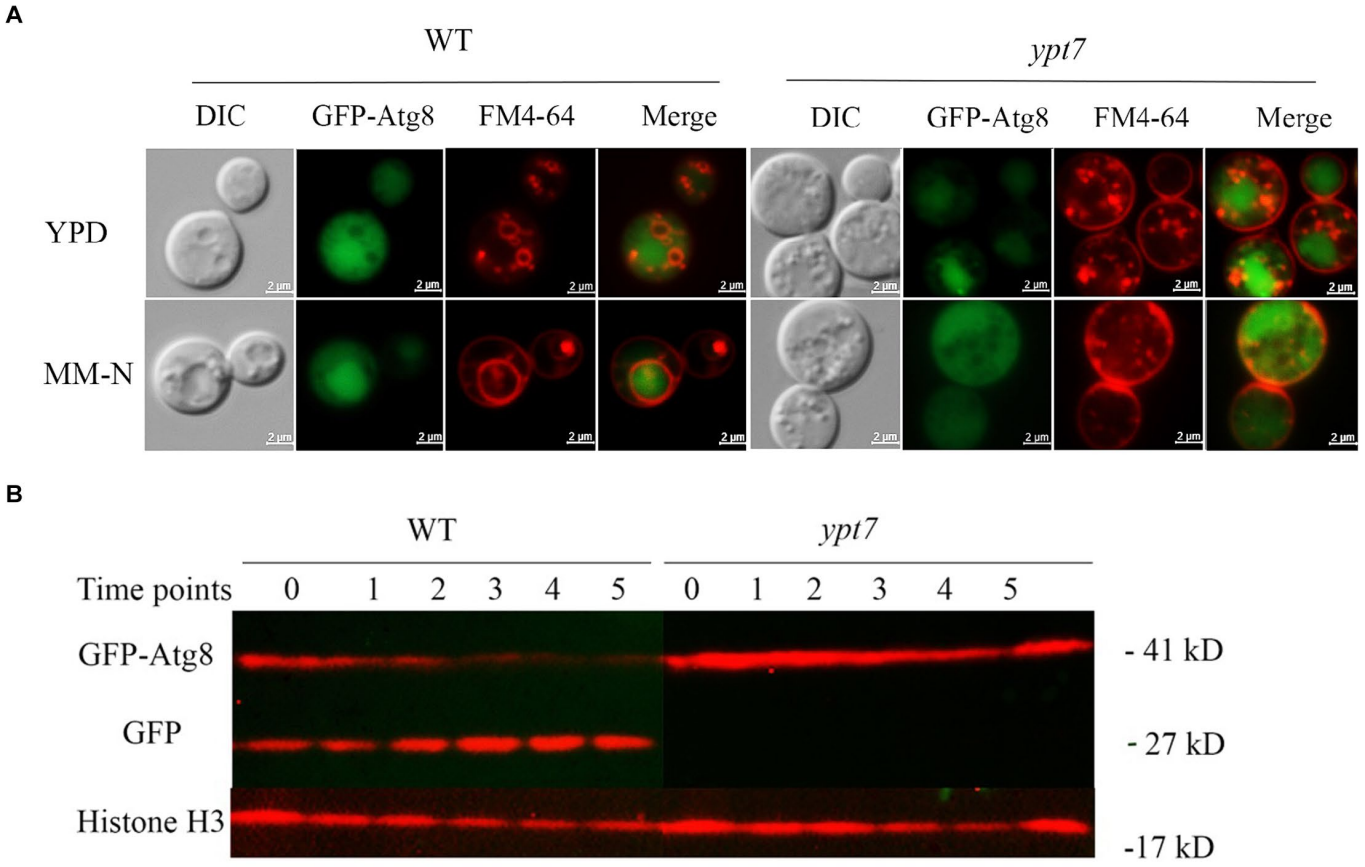
Ypt7 participates in autophagy. **(A)** Analysis of GFP-Atg8 localization in the absence of *YPT7*. The WT strain and *ypt7* deletion mutant expressing GFP-Atg8 were cultured in liquid rich medium YPD at 30°C for 24 h (upper panel) and transferred to liquid minimum medium without nitrogen source (MM-N) for 4 h (lower panel). Cells were stained with FM4-64 to reveal the vacuolar membrane, and examined under fluorescence microscopy. Bar = 2 μm. **(B)** Proteolysis of GFP-Atg8 in the WT and *ypt7* strains was analyzed by immunoblot analysis. The cells were cultured in YPD medium for 24 h, and then shifted to MM-N medium to induce the autophagy at indicated time points (hours) of nitrogen starvation. Cells were harvested and the crude protein extracts were analysed by immunoblotting using a GFP antibody. The blots were re-stripped, and probed with rabbit histone 3 antibody as a control for protein loading. The experiments were repeated at least for three times with consistent results.

The degradative process of autophagy for nutrient recycling upon starvation is dependent on fusion of autophagosomes with the vacuole, and subsequent degradation of enclosed cargo. Given the importance of Ypt7 in HOPS and endosome-vacuole fusion, and the role of the HOPS complex in autophagy, we examined the influence of Ypt7 deletion on autophagy by observing the localization of a GFP-Atg8 fusion by fluorescent microscopy. Atg8 is a ubiquitin-like protein that is conjugated to phosphatidylethanolamine (PE) and plays a role in cargo recruitment and expansion of the phagophore (Cheong and Klionsky, 2008; Ding et al., 2018; Barz et al., 2021). Our examination of GFP-Atg8 localization in WT cells from rich medium (YPD) or nitrogen starvation induction medium (MN-N) revealed that GFP-Atg8 was mainly distributed in the cytoplasm, similar to the earlier report of Roberto et al. (2020) (Figure 6A). For comparison, the cells were labelled with vacuole membrane marker FM4-64 before observation. After induction of autophagy by nitrogen starvation (cells were transferred to MM-N for 4 h), GFP-Atg8 was largely located in vacuoles, indicating that the GFP moiety of GFP-Atg8 was cleaved and accumulated in vacuoles, a typical aspect of autophagic flux. In *ypt7* mutants grown in rich medium (YPD), GFP-Atg8 fluorescence was mainly observed in the cell cytoplasm (Figure 6A). However, after 4 h of nitrogen starvation, GFP fluorescence in the mutants remained localized in the cytoplasm, indicating a defect in autophagy. In the *ypt7* deletion mutants, fragmented vacuoles were visible upon staining with FM-4-64, but the GFP-Atg8 signal was not discernable in vacuoles (Figure 6A). These observations are consistent with descriptions in other organisms in which GFP-Atg8 in autophagosomes is cleaved after the autophagic body membrane is lysed, and the GFP portion is resistant to vacuolar proteolysis and accumulates in vacuoles (Cheong and Klionsky, 2008; Liu et al., 2015; Zhao et al., 2019; Roberto et al., 2020).

We next examined GFP-Atg8 proteolysis after autophagy induction by nitrogen starvation. The full-length GFP-Atg8 protein (~ 41 kDa) and a cleaved, free GFP band (~ 27 kDa) were detected with in WT cells with anti-GFP antibodies upon nitrogen starvation. The level of free GFP signal increased with time of induction (up to 5 h), and at the expense of the full-length GFP-Atg8 (Figure 6B), suggesting a normal autophagic flux as described previously (Roberto et al., 2020). In contrast, only the full-length GFP-Atg8 band was detected in the cells of the *ypt7* mutant after nitrogen starvation (up to 5 h), and the full-length GFP-Atg8 signal did not decrease with induction time (Figure 6B), indicating that GFP-Atg8 proteolysis was prevented in the mutant. Taken together, these assays indicated that deletion of *YPT7* impaired autophagy.

### 3.8 Ypt7 is required for robust growth on heme as an iron source

**Figure 7:**
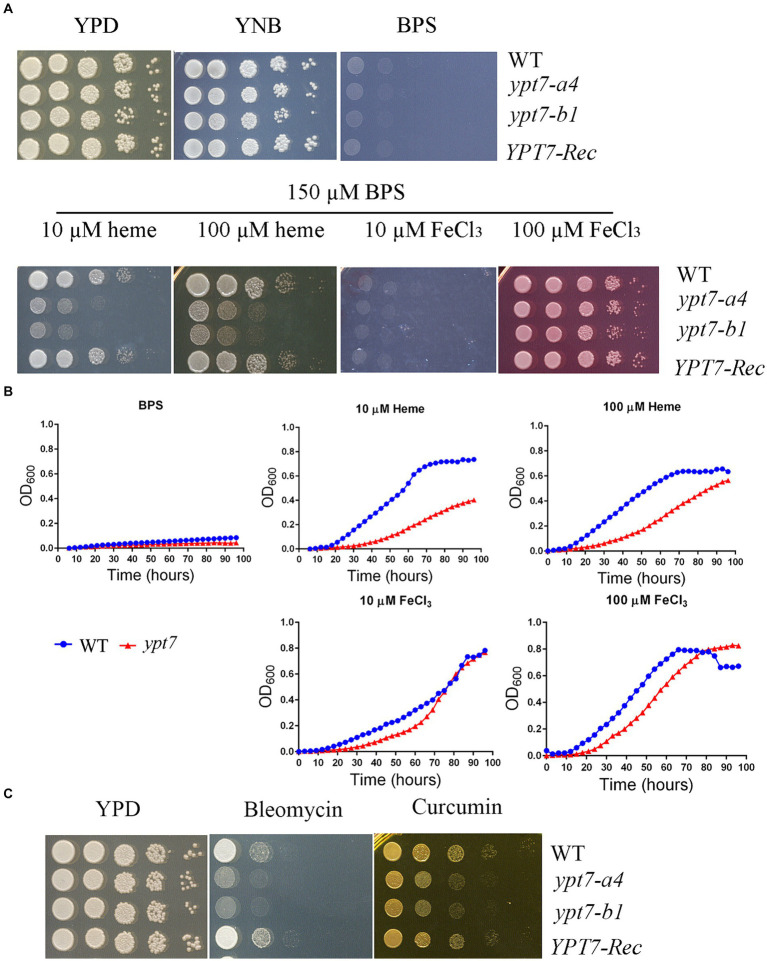
Ypt7 is required for robust growth on heme. **(A)** The growth of the WT, two independent *ypt7* mutants and the complemented strain was tested on YPD, YNB and YNB plus BPS supplemented with either heme or FeCl_3_ at 10 μM or 100 μM at pH 7.0. Ten-fold serial dilutions of each strain were spotted on the indicated media, and the plates were incubated at indicated temperatures for 2 days before being photographed. **(B)** Cells of the WT and *ypt7* mutant (*ypt7-a4*) strains were inoculated into liquid YNB medium plus 150 μM BPS without and with supplementation with either heme or FeCl_3_ at 10 μM or 100 μM at pH 7.0. The cultures were incubated at 30°C, and OD_600_ was measured. The assays were repeated at least three times. Data represent the averages from three independent experiments ± standard deviations (SD). Statistical significance was determined by ANOVA in GraphPad V6, and the difference in growth between WT and *ypt7* strains on either 10 or 100 μM of heme is significant (*p* < 0.001). **(C)** Ypt7 influences sensitivity to bleomycin and curcumin. Ten-fold serial dilutions of each strain were spotted on media with the indicated drugs, and the plates were incubated at 30°C for 2 days before being photographed.

Previously we found that functions related to endocytosis and endosomal trafficking, including ESCRT components, CORVET and HOPS complexes, are required for iron use from heme and inorganic iron sources in *C. neoformans* (Hu et al., 2013, 2015; Caza et al., 2018; Bairwa et al., 2019; Hu et al., 2021a). We therefore investigated the contributions of Ypt7 to growth on various iron sources because of the role of the protein in endocytosis. The strains were first grown for 2 days in yeast nitrogen base-low-iron medium (YNB-LIM, i.e., YNB containing BPS) to exhaust intracellular iron stores, and growth was then tested on YNB-LIM at neutral pH (pH 7.0), without or with FeCl_3_ or heme (Figure 7A). All strains grew robustly on iron-replete YNB medium, but failed to grow on iron-depleted medium (YNB-LIM). The WT strain also grew on YNB-LIM with the addition of heme or FeCl_3_ (10 or 100 μM). The *ypt7* mutants grew like WT on the medium with either 10 or 100 μM FeCl_3_, but showed reduced growth on YNB-LIM supplemented with heme at either 10 or 100 μM at pH 7.0 (Figure 7A). Growth assays were also performed in liquid YNB-LIM for all strains with similar results, although a more significant growth defect of the *ypt7* mutant at 10 μM of heme was noted (Figure 7B). Overall, these assays indicated that Ypt7 is required for growth on heme as the sole iron source. Additional growth assays were performed with the medium supplemented with either bleomycin, an inducer of DNA breaks which is dependent on iron, or curcumin, an iron chelator. These reagents have been used previously to assess the iron-related phenotypes in fungi (Jung et al., 2006; Hu et al., 2017). As shown in Figure 7C, deletion of *YPT7* impaired growth on medium supplemented with either bleomycin or curcumin. These results provide additional evidence that Ypt7 plays an important role in iron homeostasis, although the underlying mechanisms required further investigation.

**Figure 8:**
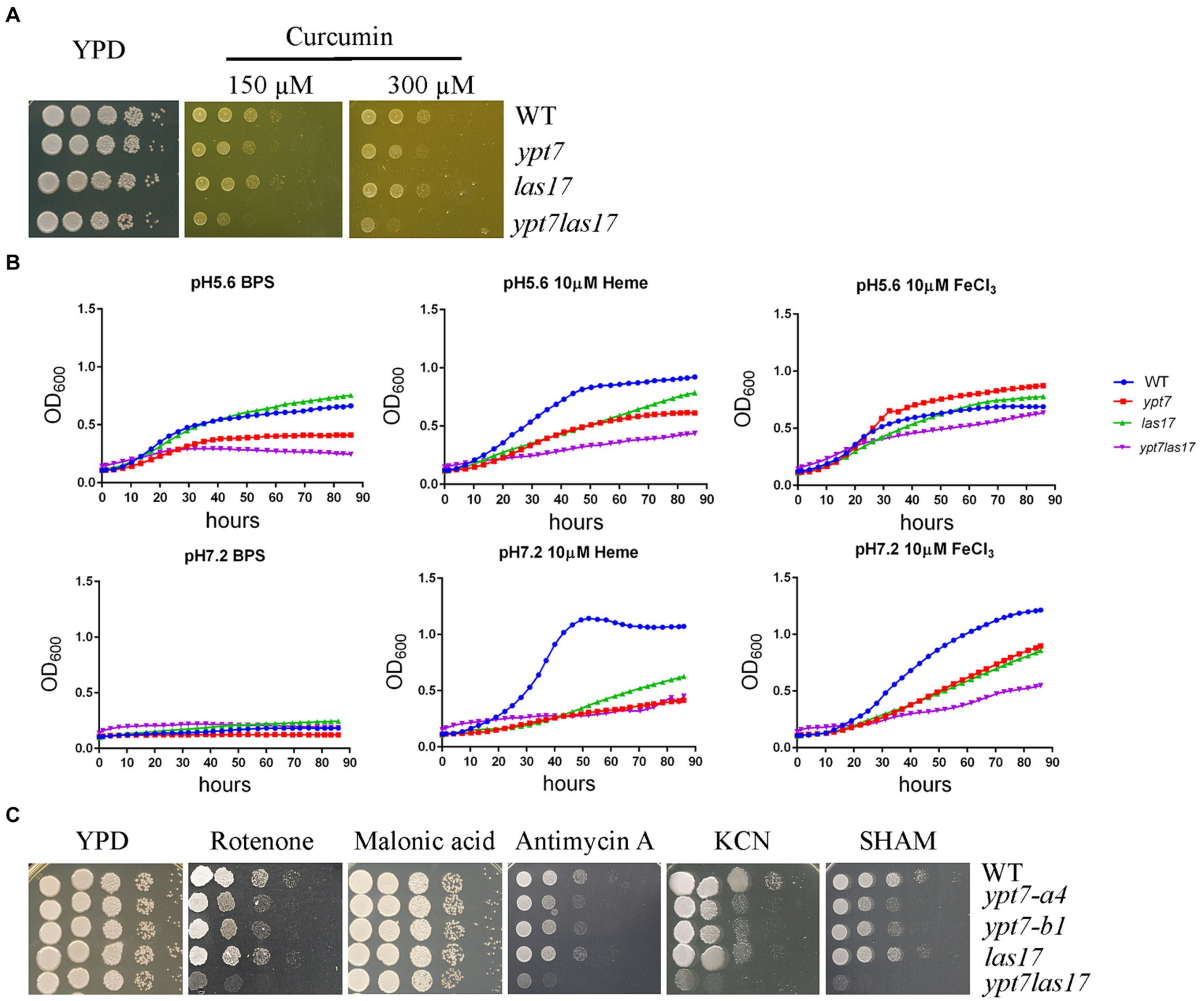
Loss of both Ypt7 and Las17 has an additive impact on iron utilization and mitochondrial functions. **(A)** The growth of the WT, two independent *ypt7* mutants and the complemented strain was tested on YPD supplemented with curcumin at the indicated concentration. Ten-fold serial dilutions of each strain were spotted on the indicated media, and the plates were incubated at indicated temperatures for 2 days before being photographed. **(B)** Cells of the WT, *ypt7* mutant (*ypt7-a4*), *las17*, and *ypt7 las17* double deletion mutant strains were inoculated into liquid YNB medium plus 150 μM BPS with and without supplementation with either heme or FeCl_3_ at 10 μM at pH 5.6 or pH 7.2. The cultures were incubated at 30°C, and the OD_600_ was measured. The assays were repeated for at least three times. Data represent the averages from three independent experiments ± standard deviations (SD). Statistical significance was determined by ANOVA in GraphPad V6. **(C)** Spot assays of WT, *ypt7* mutant (*ypt7-a4*), *las17*, and *ypt7 las17* double deletion mutant strains on YPD supplemented with the indicated ETC inhibitors. Ten-fold serial dilutions of each strain were spotted on media with the indicated drugs, and the plates were incubated at 30°C for 2 days before being photographed.

We previously demonstrated a role for clathrin-mediated endocytosis (CME) in the uptake and trafficking of heme in *C. neoformans* (Bairwa et al., 2019). For example, Las17, one CME component is required for optimal growth on low-iron media containing hemin as an iron source in *C. neoformans* (Bairwa et al., 2019). To explore the relationship between CME and Ypt7 for iron acquisition, we deleted *LAS17* in a *ypt7* mutant and tested the iron-related phenotypes of the resulting *ypt7 las17* double mutant. Deletion of both *LAS17* and *YPT7* resulted in a more marked growth defect compared to each single mutant on medium supplemented with curcumin, an iron chelator, suggesting an additive role in iron utilization (Figure 8A). We also examined the growth of the strains in liquid iron limiting medium (YNB + BPS) and/or in YNB + BPS supplemented with either heme or FeCl_3_ as the sole iron source. We tested the growth of the strains at acidic pH of 5.6 and at physiological pH of 7.2, respectively. In iron limiting medium (YNB + BPS) at pH 5.6, the WT and *las17* strains showed slight growth, while either the *ypt7* or the *ypt7 las17* double mutant exhibited minimal growth; none of the strains grew at pH 7.2 (Figure 8B). The difference in growth is likely due to increased availability of iron at acidic pH (Johnson et al., 2012; Cadieux et al., 2013). At both acidic and physiological pHs, single *ypt7* or *las17* mutants, or the *ypt7 las17* double mutant strain displayed reduced growth in medium supplemented with heme as the sole iron source. However, the *ypt7 las17* double mutant demonstrated more markedly impaired growth with heme at the acidic condition, indicating an addictive influence on iron utilization from heme (Figure 8B). Furthermore, at the acidic pH, the *ypt7*, *las17* and *ypt7 las17* mutants grew at the level of WT strain in medium supplemented with 10 μL FeCl_3_, but displayed impaired growth at pH 7.2. Notedly, the *ypt7 las17* double mutant showed a greater growth defect at the physiological pH in this medium, compared to either of the single deletion mutants (Figure 8B), thus supporting the hypothesis that pH influences the iron availability, and Ypt7 and Las17 play an additive role in iron utilization. We also tested the growth of the strains on medium supplemented with ETC inhibitors to assess the influence of double deletion on mitochondrial functions. As described previously, the *las17* mutant, similar to the *ypt7* mutant, showed impaired growth on medium with ETC inhibitors including rotenone, antimycin, KCN and SHAM (Figure 8C). However, the *ypt7 las17* double mutant displayed a greater defect in growth compared to either of single mutant (*ypt7* or *las17*), indicating additional roles of Ypt7 and CME beyond the influence on iron utilization.

We further expressed a codon optimized version of a genetically encoded heme sensor (Bairwa et al., 2020) in both the WT strain and the *ypt7* mutant, and examined the response of the sensor to low iron and low iron with supplement of different concentrations of heme by both flow cytometry and fluorescent microscopy. Cells of the WT^hs^, and *ypt7^hs^* stains were grown in YPD, or YNB + BPS, or YNB + BPS supplemented with 100 μM heme for 3 h before flow cytometry. Compared with the cells grown in rich YPD medium, cells harvested from the low iron condition exhibited elevated ratios of GFP/mKate signal, but those from the YNB + BPS medium with addition of heme at 100 μM displayed reduced ratios, in both WT and *ypt7* strains. This finding indicates that the sensor is responsive to exogenous heme. However, we did not observe significant differences in ratios of GFP/mKate signal in cells grown in all media tested between WT and the *ypt7* mutants suggesting that deletion of *YPT7* did not cause changes in cytosolic heme levels (Supplementary Figure S5).

### 3.9 Loss of Ypt7 influences virulence factors, reduces survival in macrophages, and attenuates virulence in mice

**Figure 9:**
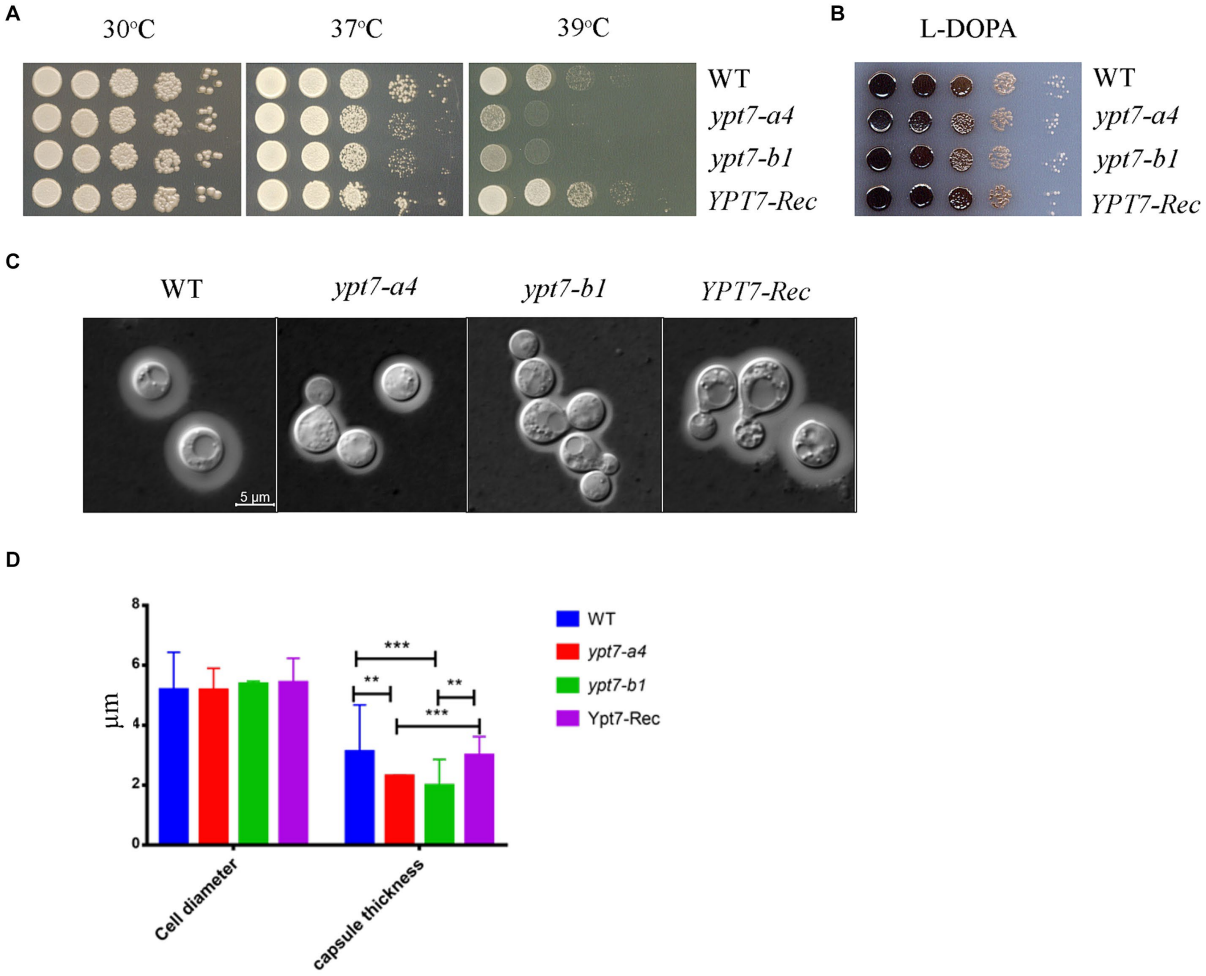
Ypt7 influences thermotolerance and capsule formation. **(A)** Serial 10-fold dilutions of the indicated strains were spotted on YPD plates. The plates were incubated at indicated temperature for 2 days before being photographed. **(B)** Melanin production was tested after growth at 30°C for 2 days by spotting serial 10-fold dilutions of the indicated strains onto L-3,4-dihydroxyphenylalanine (L-DOPA) plates. **(C)** Cells were grown in defined low-iron medium at 30°C for 48 h, and capsule formation was assessed by India ink staining for the indicated strains. **(D)** Measurement of cell diameter and capsule thickness of the WT, two *ypt7* independent mutants and the complemented strains. Cells were cultured in capsule induction media for 48 h before the measurement. At least 100 cells for each strain were measured. Statistical significance was determined by ANOVA (^***^p < 0.001 and ^**^p < 0.01) in GraphPad V6.

Next, we examined the virulence-related phenotypes in the *ypt7* mutants including growth at elevated temperatures, production of melanin, and formation of the polysaccharide capsule (Figure 9). We first observed that deletion of *YPT7* resulted in slightly (37°C) or strongly (39°C) impaired growth compared with 30°C, a phenotype similar to the HOPS *vam6* mutant (Figure 9A). Melanin formation was not influenced by loss of Ypt7, but we observed reduced production of capsule in the *ypt7* mutants, with smaller capsule size compared with the WT strain (Figures 9B–D). Overall, the absence of *YPT7* caused defects in thermotolerance and the elaboration of capsule, a major virulence factor.

**Figure 10:**
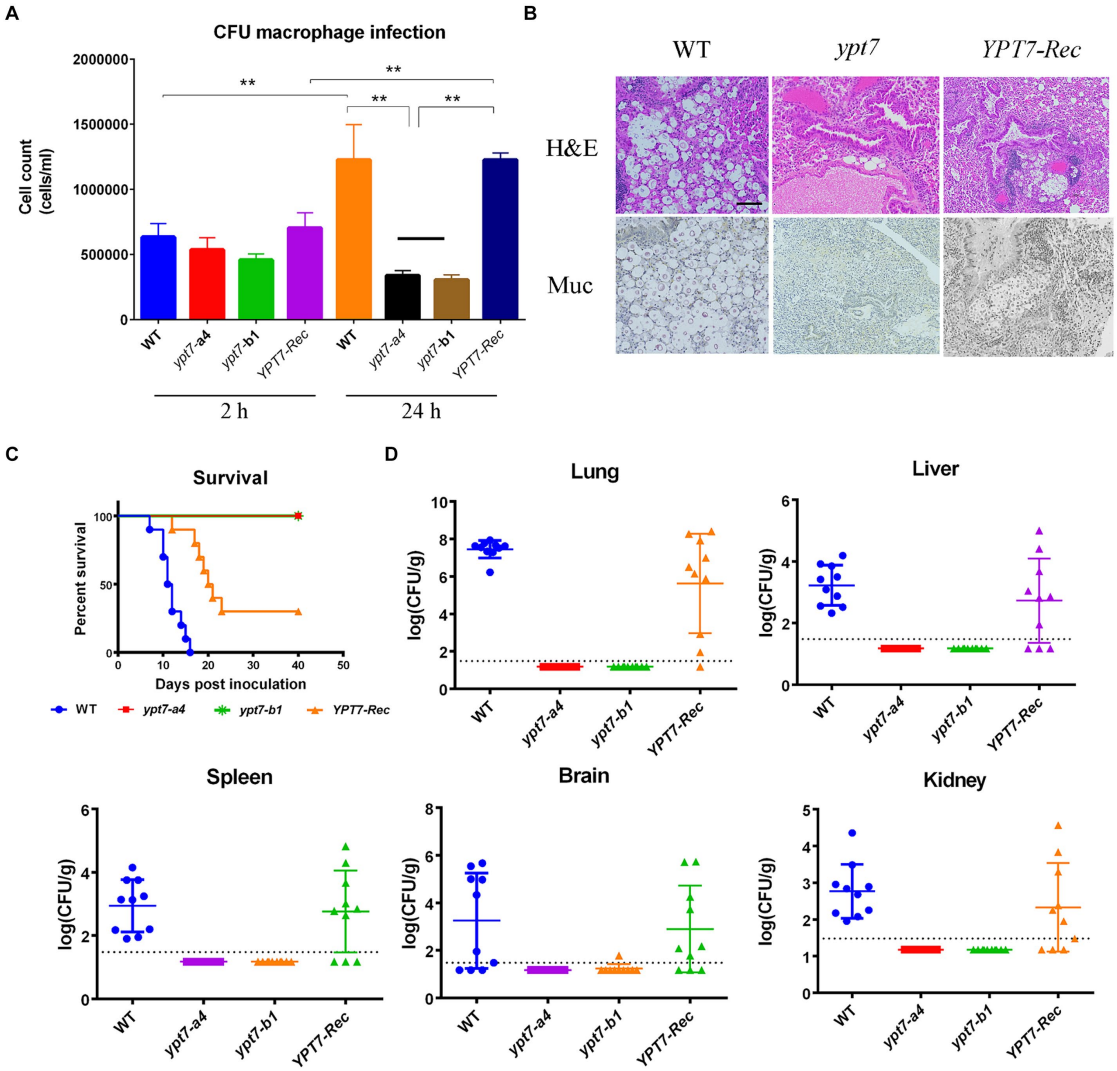
Ypt7 is required for survival in macrophages and for virulence in mice. **(A)** Cells of the wild type (WT) strain, two *ypt7* mutants, and the *YPT7* complemented strain at 2.5 × 10^5^ cells were incubated with macrophage J774A.1 cells and the wells were washed after 2 h of incubation to remove extracellular yeast cells. Fungal survival was measured by plating on YPD and counting colony-forming units (CFUs) after 2 h and 24 h of incubation. The data represent the mean values ± standard error of the mean of three independent biological experiments done in triplicate. Statistical analysis was performed using an unpaired two-tailed student’s *t*-test to determine the difference between the WT strain, two *ypt7* mutants, and the *YPT7* complemented strains separately (^**^p < 0.01). **(B)** Histopathology of lung tissue after infected with the WT, *ypt7* mutant and the complemented strains. Thin sections of pulmonary tissue from the infected mice at the humane end point of the experiment, stained with hematoxylin and eosin (H&E) or mucicarmine (Mur). Fungal cells were not observed in the *ypt7* infected mice. Bar = 100 μm. **(C)** Ten female BALB/c mice were inoculated intranasally with 2 × 10^5^ cells of each of the strains indicated, and the survival of the mice was monitored daily. Survival differences between groups of mice were evaluated by log-rank tests. The *p* values for the mice infected with the WT and mutant strains were statistically significantly different (*p* < 0.001). **(D)** Fungal burden was determined in organs (lung, brain, liver, kidney, and spleen) for all mice infected with the strains at the end of the experiment. The Mann–Whitney *U* test was used for statistical analysis. Differences in the fungal loads between the WT and *ypt7* mutants in each organ examined were statistically significant (*p* < 0.001).

The ability of fungal cells to withstand multiple stresses upon phagocytosis is an important virulence attribute (de Jesus-Berrios et al., 2003; Missall et al., 2006). The defects in virulence factor formation, autophagy, intracellular trafficking and secretion, and iron acquisition in *ypt7* mutants suggested that Ypt7 would be important for survival during interactions with macrophages, and for virulence in a mouse inhalation model of cryptococcosis. To test this idea, we examined the survival of the *ypt7* mutants during interactions with the murine macrophage-like cell line, J774A.1. As shown in Figure 10A, the cell numbers of two independent *ypt7* mutants recovered after interaction with J774.1 cells were significantly lower than those of the WT and the reconstituted strains, indicating a reduced ability to survive and proliferate. We note that inoculation of the strains into macrophage-free DMEM did not result in poor growth in the medium.

Finally, we employed a mouse inhalation model to compare the ability of the *ypt7* mutants, and the WT and the *YPT7* reconstituted strains, to impact disease and survival. In contrast to the WT strain, which caused a lethal disease in all mice by 17 days post-inoculation, both *ypt7* mutants showed an avirulent phenotype in this model, and the infected mice survived to the end of the experiment at day 40. The mice were asymptomatic and sacrificed at this time. The complemented strain partially restored virulence to the WT level and most of mice succumbed to the infection by 21 days post-inoculation (Figures 10B,C). Histological observation of infected lung tissue revealed that no fungal cells were observed in the organs from the mice infected with the *ypt7* mutants, while a high number of fungal cells were observed in lungs from the mice infected with either WT or complemented strains (Figure 10B). This observation was supported by enumeration of fungal loads in organs harvested from infected mice at the end of the experiment. That is, very few fungal cells were retrieved from lung, blood, kidney, liver, spleen, and brain from mice infected with the mutants, and high numbers of fungal cells were retrieved from these organs for mice infected with the WT and complemented strains (Figure 10D). Overall, these data indicate that the *ypt7* mutants were avirulent and unable to proliferate in mice and to disseminate to different organs beyond the lung.

## 4 Discussion

**Figure 11:**
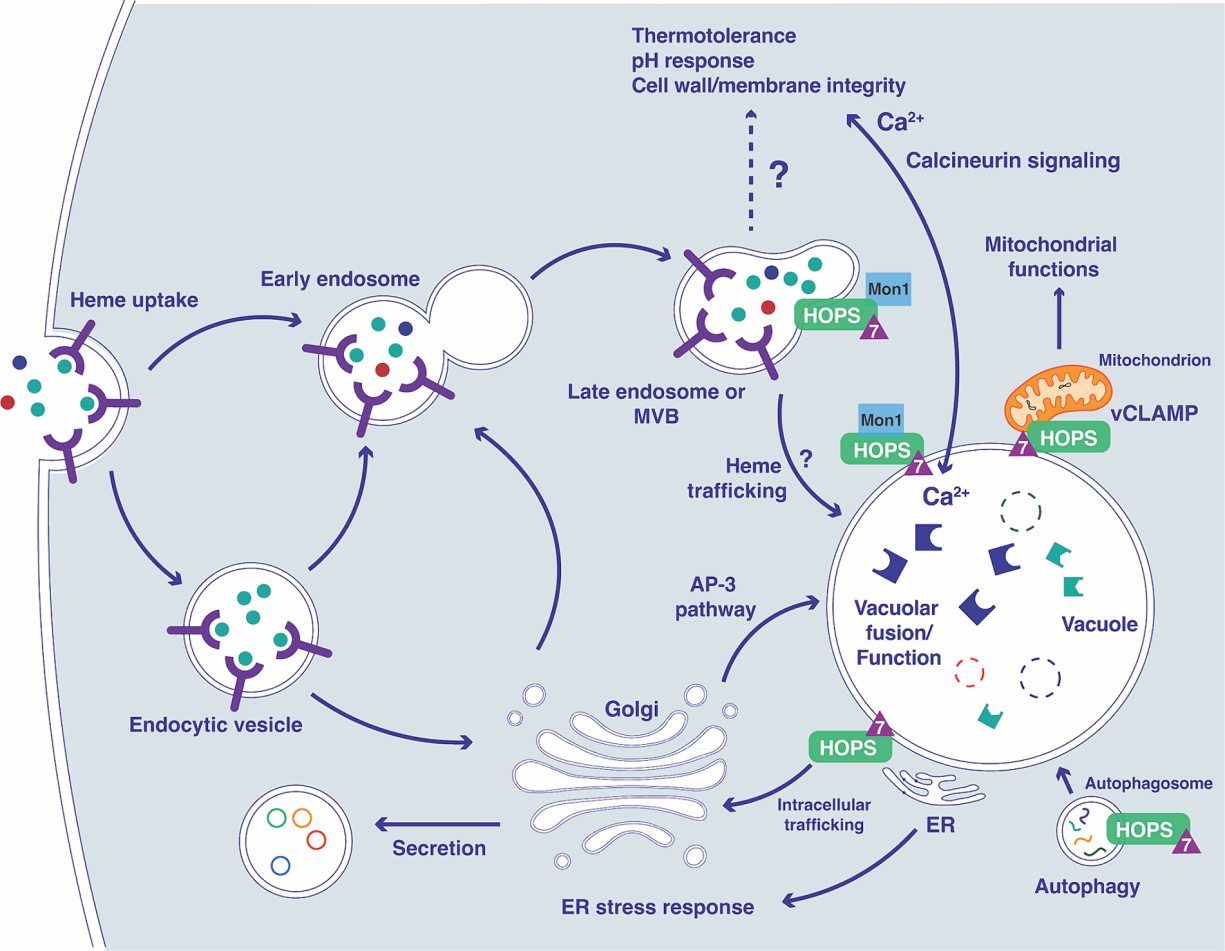
Overview of the connections between Ypt7 contributions to endomembrane trafficking, vacuolar function and phenotypes. The diagram illustrates connections between the vacuole and the endocytic pathway starting with the formation of early endosomes (containing receptors and cargo including heme) and subsequent transition to late endosomes with the participation of Ypt7 (triangle), Mon1 (Gao et al., 2018; Son et al., 2018) and the HOPS complex (Auffarth et al., 2014). The impact of loss of Vpt7 is indicated for several aspects of vacuolar function, based on phenotypes related to calcineurin signaling, mitochondrial function, autophagy, ER function, and secretion. The extensive phenotypic changes for the *ypt7* mutant contribute to impaired survival upon phagocytosis and attenuated virulence in mice. In particular, influences on calcium homeostasis and the calcineurin pathway may have a substantial impact on virulence (Park et al., 2016, 2019; Stempinski et al., 2022).

The endomembrane trafficking machinery that supports nutrient acquisition, adaptation to the host environment, and virulence is incompletely understood for fungal pathogens. In this study, we investigated the contribution of Ypt7, a Rab family GTPase, to the ability of *C. neoformans* to cause disease in vertebrate hosts. In *S. cerevisiae* and the other organisms, Ypt7 is found on late endosomes and is required for HOPS complex functions such as the homotypic fusion events in vacuole inheritance and endosome-endosome fusion (Haas et al., 1995; Borchers et al., 2021). Our analysis of mutants lacking Ypt7 revealed a conserved impact on vacuole organization and function in *C. neoformans*. Similar to loss of Vam6 and Vps41, components of the HOPS complex, *C. neoformans* mutants lacking *YPT7* exhibited fragmented vacuoles and increased sensitivity to vacuolar stressors. Additionally, a GFP-Ypt7 fusion protein localized to the vacuolar membrane and vCLAMP sites. Loss of *YPT7* in *C. neoformans* also resulted in delayed uptake of the lipophilic styryl dyes FM4-64 and MDY64, reduced secretion of acid phosphatase, and hypersensitivity to drugs (brefeldin A and monensin) that interfere with vesicle trafficking. Reduced cell-associated acid phosphatase activity and mislocalization of Aph1-DsRed in *ypt7* mutants also suggest that Ypt7 plays a role in exocytosis. These observations are consistent with the findings that Ypt7 contributes to both exocytosis and endocytic pathways in other organisms (Wichmann et al., 1992; Liu et al., 2012; Wu et al., 2021). Overall, our demonstration that Ypt7 localizes to the vacuolar membrane and regulates vacuolar functions is consistent with the functions of Ypt7 in other fungi including *S. cerevisiae*, *F. graminearum*, *A. nidulans*, and *M. oryzae* (Haas et al., 1995; Abenza et al., 2010; Liu et al., 2015; Zheng et al., 2015) (see Figure 11).

We also found that loss of *YPT7* impairs the degradation of autophagic bodies, as demonstrated by examination of GFP-Atg8. Atg8 is a critical component for the autophagy pathway and functions in membrane synthesis of the autophagosome vesicle, and delivery of cargo from the cytoplasm to the vacuole lumen (Ding et al., 2018; Roberto et al., 2020). Atg8 accumulates in the vacuole after starvation or rapamycin induction, and N-terminal GFP-tagged Atg8 has been employed to study autophagy in fungi (Mizushima, 2004; Kikuma et al., 2006; Liu et al., 2015; Roberto et al., 2020). We found that *ypt7* mutants exhibited fragmented vacuoles that lacked GFP-Atg8 under both nutrient-rich and nitrogen starvation conditions, and that loss of Ypt7 blocked GFP-Atg8 cleavage upon induction of autophagy. The involvement of Ypt7 in autophagy has been observed in other fungi including the phytopathogen, *M. grisea* and *Aspergillus nidulans* (Pinar et al., 2013; Liu et al., 2015). Ypt7 is also required for autophagosome fusion with the lysosome in mammalian cells (Gao et al., 2018; Kuchitsu and Fukuda, 2018). Furthermore, previous investigation suggests that ER plays a critical role in early autophagosome formation (Davis et al., 2017), and defective ER may impair the process. We observed that loss of Ypt7 resulted in increased sensitivity to the drug tunicamycin that induces ER stress and to brefeldin A, a trafficking stressor that also induces ER stress.

Our previous characterization of the Vam6/Vps39/TRAP1-domain proteins (Vam6 and Vps3) in *C. neoformans* revealed that both proteins contribute to robust growth on heme as the sole iron source and are required for vacuole biogenesis and endocytosis (Hu et al., 2021a). Consistent with these findings, loss of Ypt7 impaired growth on medium with heme as the sole iron source. Previously, we have found that the other proteins functioning in endosomal membrane trafficking, such as clathrin heavy chain (Chc1), ESCRT complex proteins, and Vps45, are required for the use of heme as the iron source (Hu et al., 2013, 2015, 2017; Caza et al., 2018; Bairwa et al., 2019). Additional evidence from assays on bleomycin and curcumin support the hypothesis that Ypt7 participates in iron homeostasis, although Ypt7 is dispensable for growth on inorganic iron sources at pH 5.6. An influence of pH on iron availability was noted because Ypt7 is required for optimal growth at pH 7.2 on an inorganic iron source. Loss of Ypt7 did not influence cytosolic heme levels, as detected by a heme sensor (Bairwa et al., 2020). Similarly, loss of Vam6/Vps39 in *S. cerevisiae* did not influence the kinetics of heme distribution as measured with a heme sensor (Martinez-Guzman et al., 2020). Interestingly, deletion of both *YPT7* and *LAC17* (a component of CME), impaired growth in medium with heme as the sole iron source, particularly at the acidic pH, and increased sensitivity to ETC inhibitors. These results suggested that CME and Ypt7 have distinct and shared roles in intracellular trafficking of iron and mitochondrial homeostasis, although further investigation is needed.

Similar to findings in *S. cerevisiae*, GFP-Ypt7 localized to the vacuolar membrane and vCLAMPs in *C. neoformans*, as revealed by colocalization of GFP-Ypt7 and the mitochondrial protein mCherry-Lys4, and colocalization of GFP-Ypt7 and Mitotracker Red. We noted that loss of CORVET complex proteins, by deletion of either *VPS3* or *VPS8*, did not influence the localization of Ypt7 to the vacuolar membrane. In contrast, defects in the HOPS complex, caused by deletion of either *VPS41* or *VAM6*, resulted dispersed localization of GFP-Ypt7 in the cytoplasm likely due to vacuole fragmentation. Localization of Ypt7 to vCLAMPs suggests the involvement of Ypt7 in mitochondrial dynamics and vacuolar functions. Consistent with this idea, *ypt7* mutants displayed hypersensitivity to inhibitors of the electron transport chain including rotenone, antimycin A, and SHAM. Connections between mitochondria and virulence are emerging in fungal pathogens including *C. neoformans* and *C. candida* (Duvenage et al., 2019; Horianopoulos et al., 2020; Black et al., 2021). For example, the mitochondrial protein Mrj1 functions to regulate the electron transport chain, thermotolerance, cell wall integrity and virulence in *C. neoformans* (Horianopoulos et al., 2020). Deletion of *YPT7* did not cause a growth defect with the cell wall stressor calcofluor white, but did increase sensitivity to SDS and Congo Red, suggesting that Ypt7 influences cell wall and membrane integrity. Increased staining with propidium iodide in the *ypt7* mutants after treatment with SDS indicated that Ypt7 is important for maintaining membrane integrity. This conclusion is supported by the observation that Ypt7 mutants are more sensitive to ergosterol targeting drugs, fluconazole and miconazole. In this regard, the Ypt7 ortholog Rab7 is known to influence sterol (cholesterol) trafficking in mammalian cells (van den Boomen et al., 2020; Borchers et al., 2021).

The observed inability of *YPT7* deletion mutants to survive in macrophages and to cause virulence in a mouse inhalation model of cryptococcosis is likely due to a combination of defects including impaired iron acquisition from heme, loss of virulence traits (capsule and the ability to grow at elevated temperatures), and defective vacuolar and mitochondrial functions. In particular, the ability to grow at host temperature and the elaboration of a capsule are the major virulence traits. Our study suggests that Ypt7 likely influences thermotolerance via the calcineurin signaling, including defective growth in limited or excess calcium and hypersensitivity to calcineurin pathway inhibitors (Park et al., 2019; Stempinski et al., 2022). The defect in capsule formation is intriguing and may reflect the participation of Ypt7 in exocytosis of polysaccharide, as seen with other endomembrane trafficking functions (Yoneda and Doering, 2006; Hu et al., 2013, 2015, 2017, 2021a). The loss of multiple virulence traits for the *ypt7* mutants is consistent with the observed clearance of fungal cells from all organs at the humane end of the experiment, revealing an inability to proliferate *in vivo*. These results are consistent with the observed impact of the HOPS components, Vam6 and Vps41, on the virulence in *C. neoformans* (Fan and Liu, 2021; Hu et al., 2021a). Consistent with these observations, Mon1, the putative guanine nucleotide exchange factor (GEF) subunit that activates the Ypt7 Rab GTPase is essential for vacuole trafficking, autophagy, stress survival and virulence in *C. neoformans* (Son et al., 2018). More generally, Ypt7 plays a critical role in the virulence of several plant and human pathogenic fungi including *Magneporthe grisea, F. graminearum*, and *C. albicans* (Johnston et al., 2009, 2013; Li et al., 2015; Liu et al., 2015; Zheng et al., 2015). These observations indicate that understanding the functions of Rab GTPases in fungal pathogenesis is an important area for further investigation.

## Data availability statement

The original contributions presented in the study are included in the article/Supplementary material, further inquiries can be directed to the corresponding author.

## Ethics statement

The animal study was approved by the University of British Columbia Committee on Animal Care (protocol A21-0105). The study was conducted in accordance with the local legislation and institutional requirements.

## Author contributions

GH: Conceptualization, Data curation, Formal analysis, Investigation, Supervision, Writing – original draft, Writing – review & editing. XQ: Data curation, Formal analysis, Investigation, Validation, Writing – review & editing. KB: Data curation, Formal analysis, Investigation, Validation, Writing – review & editing. PX: Data curation, Formal analysis, Investigation, Validation, Writing – review & editing. EB: Data curation, Formal analysis, Investigation, Validation, Writing – review & editing. CL: Data curation, Formal analysis, Investigation, Validation, Writing – review & editing. JK: Conceptualization, Funding acquisition, Investigation, Project administration, Supervision, Writing – review & editing.

## Footnotes

^1^ https://www.sigmaaldrich.com/deepweb/assets/sigmaaldrich/product/documents/144/558/cs0740pis-ms.pdf

## Supplementary material

SUPPLEMENTARY FIGURE S1 **(A)** Protein sequence alignment of several Ypt7 (Rab GTPase) homologues in different eukaryotic organisms. CLUSTAL W alignments of amino acid sequences for the following putative Ypt7 proteins: *Cryptococcus neoformans* var. *grubii* H99 CNAG_02575, accession ID: XP_012048028.1; *Cryptococcus gattii* WM276 CGB_K3630W, accession ID: XP_003196770.1; *Ustilago maydis* 521 UMAG_05511, accession ID: XP_011391834.1; *Puccinia triticina* 1-1 BBBD Race 1 PTTG_07916, accession ID: OAV97327.1; *Schizosaccharomyces pombe* SPBC405.04c, accession ID: O94655.1; *Candida albicans* SC5314, accession ID: XP_721474.1; *Pyricularia oryzae* 70-15 MGG_08144, accession ID: XP_003715107.1; *Saccharomyces cerevisiae* S288C YML001W, accession ID: P32939.1; *Aspergillus terreus* NIH2624 ATEG_02060, accession ID: XP_001211238.1; *Fusarium graminearum* PH-1 FGRAMPH1_01G17199, accession ID: CEF86602.1; *Mus musculus*, accession ID: CAA61797.1; *Homo sapiens*, accession ID: AAA86640.1. As indicated, the Ypt7 proteins possess the five conserved G-Box domains (G1–G5) of the Ras superfamily and the CXC motif of the Rab family. The amino acid positions are indicated as well as the extent of the homologies: asterisks denote positions which have a single, fully conserved residue and the double and single dots indicating conservation within a defined group of amino acids with a high and low score, respectively. **(B)** Phylogenetic placement of the *C. neoformans* Ypt7 protein among a selection of Ypt7 homologues. The Clustal W sequence alignment was visualized by the NCBI’s tree viewer (https://www.ncbi.nlm.nih.gov/tools/treeviewer/).

SUPPLEMENTARY FIGURE S2 Vacuolar morphology is altered in *ypt7* mutants. The cells of WT and *ypt7* mutants were stained with MDY64 and observed under the fluorescence microscopy. Bar = 5 μm. DIC, differential interference contrast.

SUPPLEMENTARY FIGURE S3 Loss of components of the HOPS or COVET complexes do not change the localization of Ypt7. GFP-tagged Ypt7 at the N-terminus was used to examine the influence of deletion of either CORVET (Vps8, Vps3) **(A)** or HOPS (Vam6, Vps41) **(B)** on localization.

SUPPLEMENTARY FIGURE S4 Co-localization of GFP-Ypt7 and MitoTracker reveals association at putative vCLAMPs. Cells of GFP-Ypt7 strain were grown at 30°C overnight before staining with MitoTracker Red CMXRos (25 nm) and observation under the fluorescence microscopy. Bar = 2 μm. DIC, differential interference contrast.

SUPPLEMENTARY FIGURE S5 Deletion of Ypt7 does change the cytoplasmic level of heme in cells. The mKATE2-cytochrome b562-eGFP fluorescent heme sensor protein (CnHS) was introduced into the *ypt7* mutant to generate the *ypt7-HS-18* strain. The changes in eGFP/mKATE2 fluorescence ratios of the CnHS in both WT^hs^ and *ypt7-HS-18* cells were measured by flow cytometry in cells incubated with hemin (100 μM) or without heme (LIM) for 60 min. The analysis was performed with a population of mKATE2-positive gated cells and is representative of three independent experiments. No statistical difference was detected between the WT^hs^ and *ypt7-HS-18* cells.
