# Supplementary material for: Loss of the putative Rab GTPase, Ypt7, impairs the virulence of Cryptococcus neoformans
Source: Front Microbiol. 2024 Jul 25;15:1437579. doi: 10.3389/fmicb.2024.1437579 (PMC11306161; doi:10.3389/fmicb.2024.1437579)
Supplement: SUPPLEMENTARY FIGURE S1 — (A) Protein sequence alignment of several Ypt7 (Rab GTPase) homologues in different eukaryotic organisms. CLUSTAL W alignments of amino acid sequences for the following putative Ypt7 proteins: Cryptococcus neoformans var. grubii H99 CNAG_02575, accession ID: XP_012048028.1; Cryptococcus gattii WM276 CGB_K3630W, accession ID: XP_003196770.1; Ustilago maydis 521 UMAG_05511, accession ID: XP_011391834.1; Puccinia triticina 1-1 BBBD Race 1 PTTG_07916, accession ID: OAV97327.1; Schizosaccharomyces pombe SPBC405.04c, accession ID: O94655.1; Candida albicans SC5314, accession ID: XP_721474.1; Pyricularia oryzae 70-15 MGG_08144, accession ID: XP_003715107.1; Saccharomyces cerevisiae S288C YML001W, accession ID: P32939.1; Aspergillus terreus NIH2624 ATEG_02060, accession ID: XP_001211238.1; Fusarium graminearum PH-1 FGRAMPH1_01G17199, accession ID: CEF86602.1; Mus musculus, accession ID: CAA61797.1; Homo sapiens, accession ID: AAA86640.1. As indicated, the Ypt7 proteins possess the five conserved G-Box domains (G1–G5) of the Ras superfamily and the CXC motif of the Rab family. The amino acid positions are indicated as well as the extent of the homologies: asterisks denote positions which have a single, fully conserved residue and the double and single dots indicating conservation within a defined group of amino acids with a high and low score, respectively. (B) Phylogenetic placement of the C. neoformans Ypt7 protein among a selection of Ypt7 homologues. The Clustal W sequence alignment was visualized by the NCBI’s tree viewer (https://www.ncbi.nlm.nih.gov/tools/treeviewer/). [file Image_1.PDF]

A

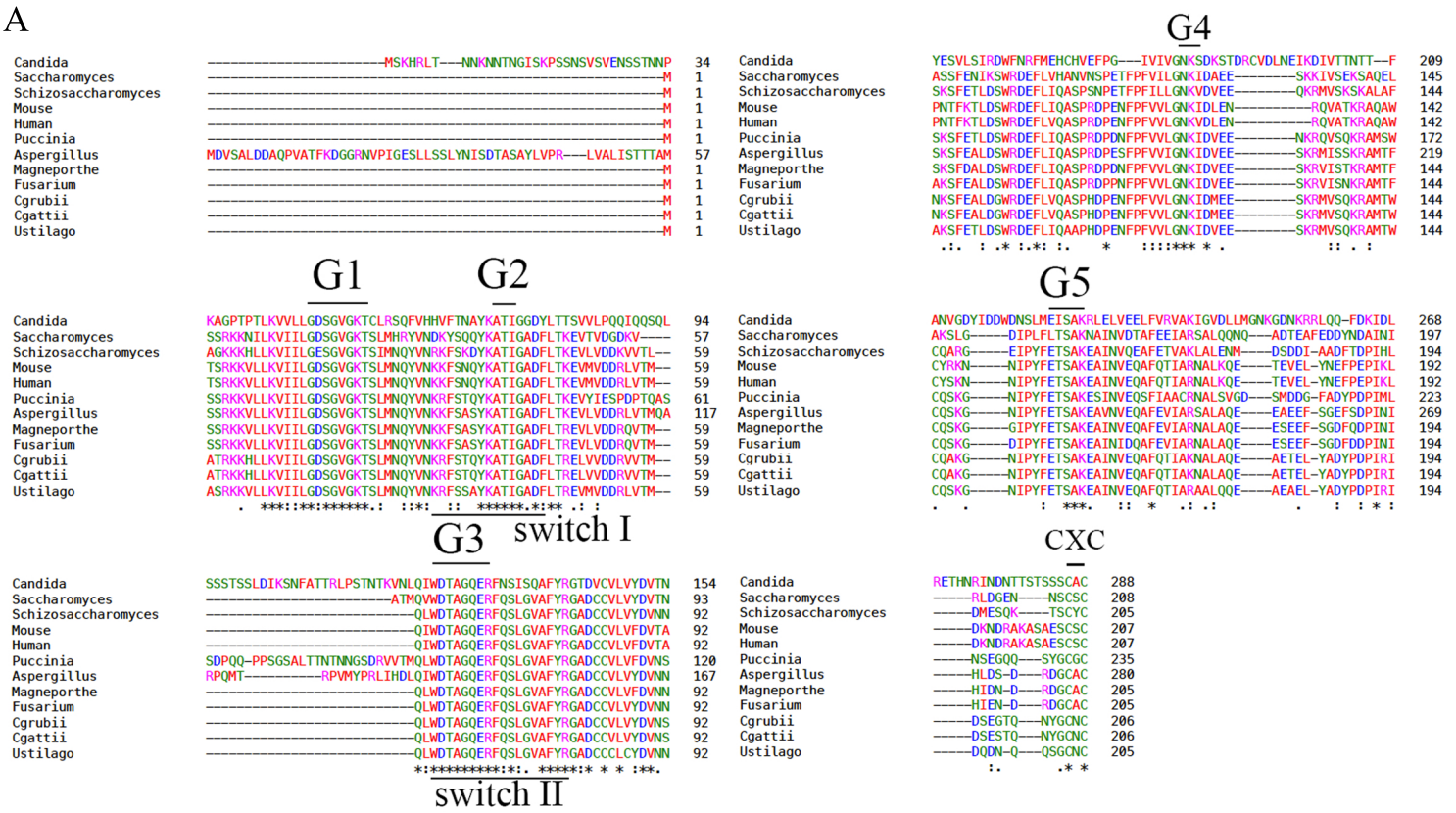
G4
G1
G2
G5
G3
switch I
CXC
switch II

B

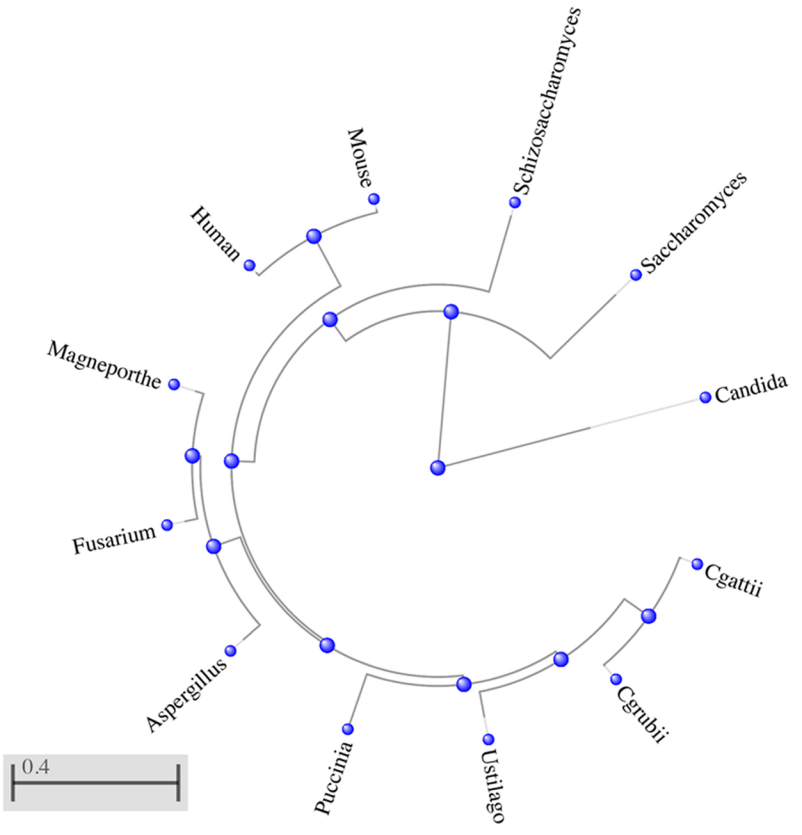
Schizosaccharomyces
Mouse
Human
Saccharomyces
Magneporthe
Candida
Fusarium
Cgattii
Aspergillus
Cgrubii
Puccinia
Ustilago
0.4
