# Supplementary figures and images for: Loss of the putative Rab GTPase, Ypt7, impairs the virulence of Cryptococcus neoformans
Source: Front Microbiol. 2024 Jul 25;15:1437579. doi: 10.3389/fmicb.2024.1437579 (PMC11306161; doi:10.3389/fmicb.2024.1437579)

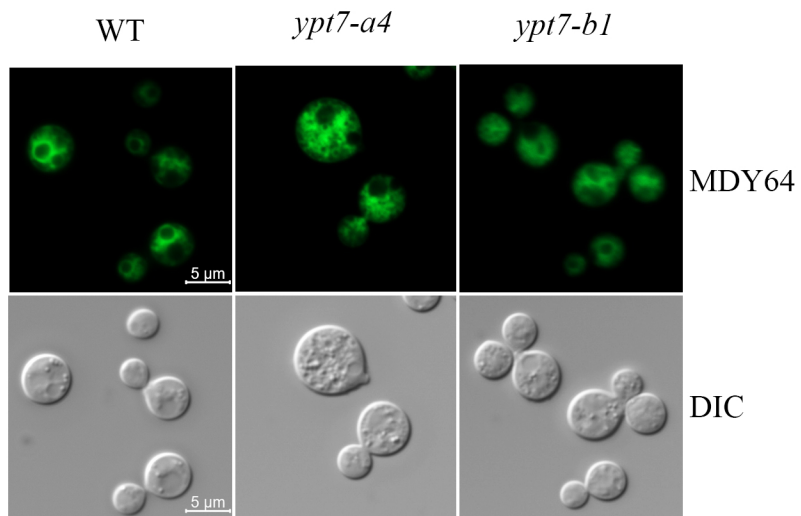

Supplement: SUPPLEMENTARY FIGURE S2 — Vacuolar morphology is altered in ypt7 mutants. The cells of WT and ypt7 mutants were stained with MDY64 and observed under the fluorescence microscopy. Bar = 5 μm. DIC, differential interference contrast. [file Image_2.PDF]

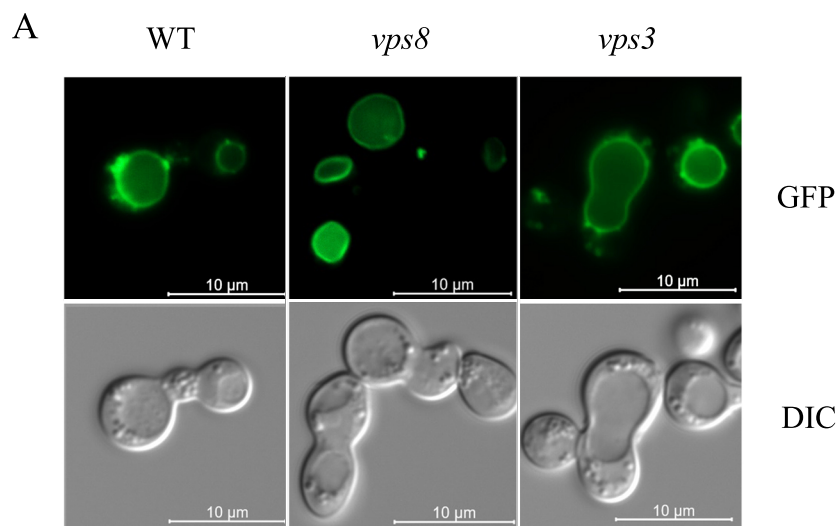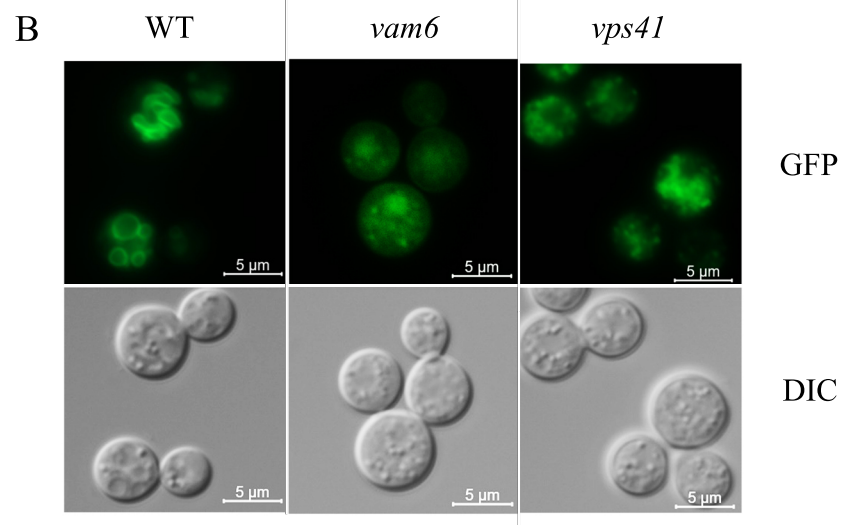

Supplement: SUPPLEMENTARY FIGURE S3 — Loss of components of the HOPS or COVET complexes do not change the localization of Ypt7. GFP-tagged Ypt7 at the N-terminus was used to examine the influence of deletion of either CORVET (Vps8, Vps3) (A) or HOPS (Vam6, Vps41) (B) on localization. [file Image_3.PDF]

DIC

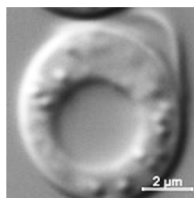

GFP-Ypt7

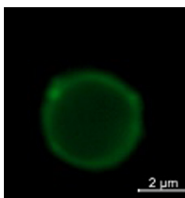

Mitotracker

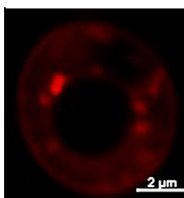

Overlay

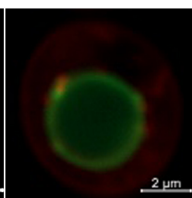

Supplement: SUPPLEMENTARY FIGURE S4 — Co-localization of GFP-Ypt7 and MitoTracker reveals association at putative vCLAMPs. Cells of GFP-Ypt7 strain were grown at 30°C overnight before staining with MitoTracker Red CMXRos (25 nm) and observation under the fluorescence microscopy. Bar = 2 μm. DIC, differential interference contrast. [file Image_4.PDF]

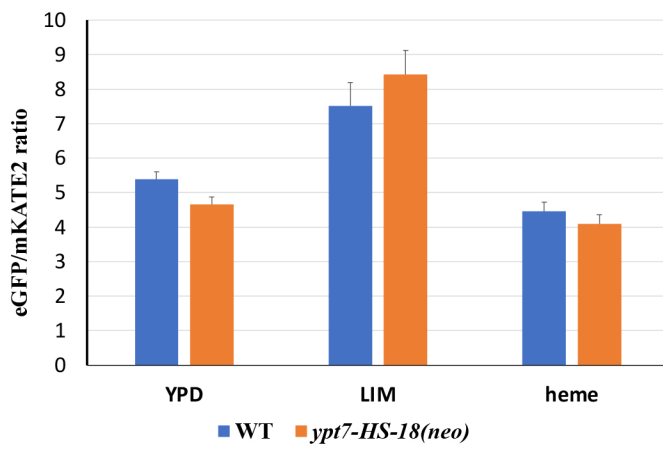

Supplement: SUPPLEMENTARY FIGURE S5 — Deletion of Ypt7 does change the cytoplasmic level of heme in cells. The mKATE2-cytochrome b562-eGFP fluorescent heme sensor protein (CnHS) was introduced into the ypt7 mutant to generate the ypt7-HS-18 strain. The changes in eGFP/mKATE2 fluorescence ratios of the CnHS in both WThs and ypt7-HS-18 cells were measured by flow cytometry in cells incubated with hemin (100 μM) or without heme (LIM) for 60 min. The analysis was performed with a population of mKATE2-positive gated cells and is representative of three independent experiments. No statistical difference was detected between the WThs and ypt7-HS-18 cells. [file Image_5.PDF]
