# Supplementary material for: Loss of the putative Rab GTPase, Ypt7, impairs the virulence of Cryptococcus neoformans
Source: Front Microbiol. 2024 Jul 25;15:1437579. doi: 10.3389/fmicb.2024.1437579 (PMC11306161; doi:10.3389/fmicb.2024.1437579)
Supplement: Supplementary file 6 [file Data_Sheet_1.pdf]

Supplementary Table S1: Strains used in the study

Strain name	Description, genotype	Reference
H99/WT	*C. neoformans* wild type strain, serotype A	Joe Heitman lab
ypt7-a4	*ypt7* deletion mutant in the H99 background, *ypt7::HYG*	This study
ypt7-b1	*ypt7* deletion mutant in the H99 background, *ypt7::HYG*	This study
GFP-YPT7 (YPT7-Rec)	A *ypt7-a4* deletion mutant strain complemented with a GFP tagged *YPT7* genomic DNA copy at the N-terminal of original *YPT7* location, *H3pro-GFP::YPT7::NEO ypt7::HYG*	This study
GFP-Ypt7 Lys4-mCherry	A *GFP-YPT7* strain in H99 background with a mCherry tagged Lys4. *LYS4::mCherry::NAT GFP::YPT7::NEO*	This study
Aph1-DsRed	A DsRed tagged Aph1 at C-terminal in the H99 strain background, *APH1::DsRed::NEO*	Lev et al., 2014
ypt7-Aph1-DsRed	*ypt7* deletion mutant in Aph1-DsRed tagged strain in the H99 background, *ypt7:: HYG APH1::DsRed::NEO*	This study
vam6-Aph1-DsRed	*vam6* deletion mutant in Aph1-DsRed tagged strain in the H99 background, *vam6:: HYG APH1::DsRed::NEO*	This study
vps41-Aph1-DsRed	*vps41* deletion mutant in Aph1-DsRed tagged strain in the H99 background, *vps41:: NAT APH1::DsRed::NEO*	This study
GFP-ATG8	Wild type H99 strain with a histone 3 promoter and GFP tagged at the N-terminal of *ATG8. GFP::ATG8::NAT*	This study
ypt7-GFP-ATG8	*ypt7* deletion mutant in the H99 background with a histone 3 promoter and GFP tagged at the N-terminal of *ATG8. GFP::ATG8::NAT ypt7::HYG*	This study

vam6	*vam6* deletion mutant in the H99 background, *vam6::HYG*	Hu et al., 2021
vps41	*vps41* deletion mutant in the H99 background, *vps41::NAT*	Hu et al., 2021
las17	*las17* deletion mutant in the H99 background, *las17::NAT*	Baiwar et al., 2018
ypt7las17	*las17* deletion mutant in *ypt7* mutant in the H99 background, *las17::NAT ypt7::HYG*	This study

Supplementary Table S2. Primers used in the study

Primer name	Primer sequence (5'->3')
Ypt7-P1	CATCTCTTCCGCTCTTTCGTTCATCTTATC
Ypt7-P2	TGCGTCAGTTTAGTGCCCGGACATTGCTGCGAGGATGTGAGCT
Ypt7-P3	AGCTCACATCCTCGCAGCAATGTCCGGGCACTAAACTGACGCA
Ypt7-P4	TAGTTTCTACATCTCTTCCTCCCTATTCCATGACCCCGACTCTCT
Ypt7-P5	AGAGAGTCGGGGTCATGGAATAGGGAGGAAGAGATGTAGAAACT A
Ypt7-P6	CCTTCAGTGTTATCGCTTGCCTGACAGCT
Ypt7-P7-NE	CAAGGGTCAAGGCGTCATAGC
Ypt7-P8-NE	AGTGACAACCCTATCATCAACCACCAAC
Hyg-PO-L	GCCACTCGAATCCTGCATGCTTATGTGAGT
Hyg-PO-R	CAGCAACGCCGTTGAATCCTCAGGATCTTCA
Ypt7-P9-PO	CTCTGGCGGCTCAACCTCTTCA
Ypt7-P10-PO	CCGCCATGAGAGTGTGCGTGTTCCTGCT
Ypt7-pro-L1	CATCTTATCCAACCATCTCTGCAACA
Ypt7-pro-L2	TCTCTTCCTCTTCCTTGAACCCCT
Ypt7-pro-R	GCTCACATCCTCGCAGCAGTGAATGAATGTTGTGAATGTTGTCA
P-Neo-marker-L	TGACAACATTCACAACATTCATTCACTGCTGCGAGGATGTGAGCT
P-Neo-marker-R	TCTGCCGAGCTCGGTACCCGGGGATCCTCGAAGAGATGTAGAAA CTA
H3GFP-L	TAGTTTCTACATCTCTTCGAGGATCCCCGGGTACCGAGCTCGGCA GA
H3GFP-R	AGATGCTTCTTCCTGGTAGCCATCTTGTACAGCTCGTCCATGCCGT GAGTGA
P-GFP-Ypt7-L	TCACTCACGGCATGGACGAGCTGTACAAGATGGCTACCAGGAAG AAGCATCT
P-GFP-Ypt7-R	CGACTCACAATTCGGTCTCAGCTTCT
Ypt7GFP-screen-5R	CACATGGTCCTGCTGGAGTTCGTGAC
Ypt7GFP-screen-3L	CGGCATACCTGGCCACATTGTACATTGA
GFP-Atg8-P1L	AGGGTGATAGTATAGAGTGATGGATC
GFP-Atg8-P1R	CCAGCTCACATCCTCGCAGCTACTGAAATAGGTGGGATGTGATAG T
GFP-Atg8-P2L	ACTATCACATCCCACCTATTTCAGTAGCTGCGAGGATGTGAGCTG G
GFP-Atg8-P2R	TCTGCCGAGCTCGGTACCCGGGGATCCTCAGAAGAGATGTAGAA ACTAG
GFP-Atg8-P3L	CTAGTTTCTACATCTCTTCTGAGGATCCCCGGGTACCGAGCTCGG CAGA
GFP-Atg8-P3R	TCATCCTTAAACTTGCTTCGGACCATCTTGTACAGCTCGTCCATGC CGTGAGTG

GFP-Atg8-P4L	CACTCACGGCATGGACGAGCTGTACAAGATGGTCCGAAGCAAGTTTAAGGATGA
GFP-Atg8-P4R	ATTGTTCAAGGTCGCCAAAGGTG
GFP-Atg8-P1La	CAGCTTATCTGAGACTGATGGTGGGAC
GFP-Atg8-P4Ra	CAACAGTATTATCGTACTGCCT
Nat-3L-long	GCCACTCGAATCCTGCATGCTTATGTGAGT
Nat-5R-long	CAGCAACGCCGTTGAATCCTCAGGATCTTCA
Lys4-mCh-P1F	AACAGAAACTACAAAGGTCGTATGG
Lys4-mCh-P1R	ATCCTCCTCGCCCTTGCTCACCATTAATGAAGCTCTAATGAAACCTTCCA
Lys4-mCh-P2F	TGGAAGGTTTCATTAGAGCTTCATTAATGGTGAGCAAGGGCGAGGAGGAT
Lys4-mCh-P2R	TTCGCCCTCAAGAATATAGTTTAGCGCTGCGAGGATGTGAGCTGGAGAG
Lys4-mCh-P3F	CTCTCCAGCTCACATCCTCGCAGCGCTAAACTATATTCTTGAGGGCGAA
Lys4-mCh-P3R	CTTGTCCAATCATCTTCATCACCAG
Las17-P7-NE	TCTCAGGCCATGAAACAGTTTAT
Las17-P8-NE	TTGCCAGCTTGGGTCAACGCCAGT
Las17-P9-PO	GTACGTAGTAGTAGTTATATACT
Las17-P10-PO	CCCTAACCTCCGTCCTCAAAGAAA
Las17-W1	CGGTGCATTAGGTTTCAAGCAG
Las17-W6	GAGGACGCTGAACTCTTATCTT

Supplementary Table S3. Gene nomenclature for Rab/Ytp GTPases

S. cerevisiae	Human	*Candida albicans*		*Ustilago maydis*		*Cryptococcus neoformans*	
Protein(s)	Equivalences	Protein	#FungiDB	Protein	#FungiDB	Protein	#FungiDB
Ypt52/Ypt53	RAB5	Ypt52<br>Ypt53	C1_14100W_A<br>C1_05350W_A	Rab5a	UMAG_10615	Rab51	CNAG_06114
Vps21 (Ypt51)	RAB5a,b,c	Vps21	CR_08060C_A	Rab5b	UMAG_02485	Rab5	CNAG_04771
Ypt7	RAB7	Ypt72<br>Ypt7	C2_06910W_A<br>C1_02870W_A	Rab7	UMAG_05511	Ypt7	CNAG_02575
Ypt1	RAB1a,b	Ypt1	C1_03500W_A	n.s.	UMAG_03833	n.s.	CNAG_05068
Ypt31/32	RAB11a, b	Ypt31	CR_07520C_A	Rab11	UMAG_01651	n.s.	CNAG_02367
-	-	-	-	n.s.	UMAG_11007	(RabX)	CNAG_04655
-	RAB2a,b	-	-	n.s.	UMAG_05212	n.s	CNAG_00875
-	RAB4a,b,c	-	-	Rab4	UMAG_01735	n.s	CNAG_02289
Ypt6	RAB6a,b,c	Ypt6	CR_06520C_A	n.s.	UMAG_11205	n.s	CNAG_06049
Sec4	RAB8a,b	Sec4	CR_01750C_A	n.s.	UMAG_03865	Sec4 (Sav1)	CNAG_02817
-	RAB18	-	-	n.s.	UMAG_03602	-	-
Ypt10	-	-	-	-	-	-	-
Ypt11	-	-	-	-	-	-	-
